# Iron Promotes Dihydroartemisinin Cytotoxicity *via* ROS Production and Blockade of Autophagic Flux *via* Lysosomal Damage in Osteosarcoma

**DOI:** 10.3389/fphar.2020.00444

**Published:** 2020-05-05

**Authors:** Ying Shen, Bin Zhang, Yanwei Su, Shaikh Atik Badshah, Xiaofei Wang, Xin Li, Yanru Xue, Li Xie, Zhe Wang, Zhouqi Yang, Ge Zhang, Peng Shang

**Affiliations:** ^1^Research and Development Institute in Shenzhen, Northwestern Polytechnical University, Shenzhen, China; ^2^School of Life Science, Northwestern Polytechnical University, Xi’an, China; ^3^Key Laboratory for Space Bioscience and Biotechnology, Institute of Special Environment Biophysics, School of Life Science, Northwestern Polytechnical University, Xi’an, China; ^4^Biomedical Experimental Center, Xi’an Jiaotong University, Xi’an, China; ^5^Law Sau Fai Institute for Advancing Translational Medicine in Bone & Joint Diseases (TMBJ), Hong Kong Baptist University, Hong Kong, Hong Kong

**Keywords:** autophagy, reactive oxygen species, iron, cancer, dihydroartemisinin, lysosome

## Abstract

Osteosarcoma cellular iron concentration is higher than that in normal bone cells and other cell types. High levels of cellular iron help catalyze the Fenton reaction to produce reactive oxygen species (ROS), which promotes cancer cell proliferation. Dihydroartemisinin (DHA), a classic anti-malarial drug, kills plasmodium through iron-dependent ROS generation. In this research, we observed the anti-osteosarcoma effects and mechanisms of DHA. We found that DHA induced ROS production, caused mitochondrial damage, and activated autophagy *via* stimulation of the ROS/Erk1/2 pathway. As the storage site for a pool of ferrous iron, lysosomes are often the key organelles affected by drugs targeting iron. In this study, we observed that DHA induced lysosomal superoxide production, leading lysosomal membrane permeabilization (LMP), and autophagic flux blockage. By reducing or increasing cellular iron using deferoxamine (DFO) or ferric ammonium citrate (FAC), respectively, we found that DHA inhibited osteosarcoma in an iron-dependent manner. Therefore, iron may be a potential adjuvant for DHA in osteosarcoma treatment.

## Introduction

Iron is an essential element that plays crucial roles in various bioprocesses in bone (Batata et al., 1967; McGinnis et al., 1979; Harrison et al., 1996; Tsay et al., 2010). It is now well established that iron regulates the balance of bone metabolism (Canali et al., 2017; Cheng et al., 2017; Jeney, 2017). In previous studies, we found that iron overload-induced bone loss while iron deficiency was associated with low bone density (Jia et al., 2014; Zhang et al., 2014; Yang et al., 2017). Osteosarcoma, the typical type of bone tumor, is strongly iron-philic to meet the needs of its abnormally-high proliferation rate of tumors (Raza et al., 2014). Thus, osteosarcoma cells often have a high intracellular iron concentration. However, high intracellular iron content is a double-edged sword because intracellular ferrous iron catalyzes the Fenton reaction to produce reactive oxygen species (ROS), placing cancer cells under a fragile redox balance (Andrews, 2000). This high intracellular iron level may be exploited as an anti-tumor therapeutic, using certain drugs that specifically target iron and the redox system (Basuli et al., 2017; Bajbouj et al., 2018).

The antimalarial and antiproliferative drug dihydroartemisinin (DHA), derived from artemisinin, has been reported to function by interacting with free iron in the food vacuole of the malaria parasite, leading to damage of the vacuole through lethal ROS generation (Antoine et al., 2014; Dalal and Klemba, 2015). In addition to antimalarial effects, DHA has been found to also exhibit actions against cancer (Mao et al., 2013; Qin et al., 2015; Zou et al., 2019), inflammatory bowel disease (Yan et al., 2019), alcoholic fatty liver (Chen et al., 2019), osteoarthritic synovium (Li D. et al., 2019), lipopolysaccharide-induced osteoclastogenesis and bone loss (Dou et al., 2016), liver fibrosis (Zhang et al., 2017), and systemic lupus erythematosus (Li Y. et al., 2019). Especially, DHA effectively inhibits cancer cell proliferation *in vitro* and *in vivo* (Liao et al., 2014). Previous studies have shown that DHA induced cell death *via* multiple pathways in the breast cancer cells, human hepatocellular carcinoma cells, prostate cancer cells, leukemia cells, and ovarian cancer. Mao H et al. found that DHA induces apoptosis of the breast cancer cells *via* Bim/Bcl-2 pathway (Mao et al., 2013). Moreover, DHA promotes hepatocellular carcinoma cells apoptosis by upregulating tumor necrosis factor *via* JNK/NF-κB pathway (Wu L. et al., 2019), inhibiting the specificity protein 1 pathway (Im et al., 2018) and activating Bim-mediated intrinsic pathway (Qin et al., 2015). Also, DHA is reported to influence the autophagy of liver cancer cells through AKT-mTOR pathway suppression (Zou et al., 2019). Furthermore, in prostate cancer cells, DHA bought about apoptosis by decreasing HSP70 expression (Xu et al., 2016). DHA is found to stimulate leukemia cells ferroptosis through ferritinophagy, a type of ferritin degradation depending on autophagy (Du et al., 2019). Also, DHA can induce leukemia cell apoptosis *via* caspase activation, cytochrome c release, Mcl-1 down-regulation, and MEK/ERK inactivation (Gao et al., 2011). DHA induces ovarian cancer apoptosis *via* inhibition of the hedgehog signaling pathway (Liu et al., 2018). Thus, suggesting that DHA effects and prevents proliferation of various cancer cells through several pathways.

In mammalian cells, the lysosome containing a pool of redox-active iron functioned like vacuole in the malaria parasite (Dielschneider et al., 2017). Iron-mediated generation of ROS and superoxide results in lysosome dysfunction and lysosomal membrane permeabilization (LMP) (Boya et al., 2003). LMP favors the release of the cathepsins into the cytoplasm, and the blockade of the autophagic flux leading to cell death (Boya et al., 2003). Lysosomes contain more redox-active iron in cancer cells than in non-cancerous cells, which makes these lysosomes more susceptible to oxidative stress and certain types of drug treatments (Mai et al., 2017). However, the effects of DHA on lysosomes in osteosarcoma remain unclear.

ROS and superoxide production triggers autophagosomes formation, which degraded in lysosomes (Watson et al., 2015; He et al., 2016). LC3B is a primary marker of autophagosome formation, while p62 expression indicates the degradation of autophagosome (Gump and Thorburn, 2014). Autophagic flux can thus be assessed by measuring the levels of autophagy-related proteins like ATG14, ATG12, and ATG5. LC3B II usually indicates the upstream activation of the autophagic process, while p62 indicates the downstream degradation of autophagic vacuoles (Kabeya et al., 2000). Thus, autophagic flux is closely related to the efficiency of lysosome function (Yuan et al., 2016). The specific effect of lysosomal iron-mediated ROS production on autophagy requires further study.

It has been reported that DHA instigates iron-dependent ROS and superoxide production *via* the iron-reactive capacity of the endoperoxide bridge, resulting in the inhibition of the cancer cells growth (Liao et al., 2014). To clarify whether lysosomes with high concentrations of iron are the central target of DHA in osteosarcoma requires further research. Therefore, we observed the role of iron concentration on the anti-osteosarcoma effects of DHA, using the iron chelator deferoxamine (DFO) or the iron supplement ferric ammonium citrate (FAC) and we further explored the molecular mechanism underlying the anti- osteosarcoma properties of DHA in this research.

## Materials and Methods

### Chemicals and Antibodies

Dihydroartemisinin was obtained from Ourchem (Sinopharm Chemical Reagent Co, Ltd, China). Dimethyl sulfoxide (DMSO), cisplatin, acridine orange (AO), deferoxamine (DFO), N-acetyl-L-cysteine (NAC), and ferric ammonium citrate (FAC) were purchased from Sigma (USA). CCK-8 kit was obtained from Dojindo (Japan). EdU Imaging Kit was bought from Ribobio (China). Lysosome Staining Kit was obtained from Abnova (USA). Lysotracker probe and Mito-tracker Red probe were procured from Beyotime (China). ROS Kit and JC-1 kit were acquired from Beyotime (China). AO/EB Staining Kit was obtained from Senbeijia (China). Primary antibodies for rabbit polyclonal to LC3B (Abcam, ab48394), rabbit monoclonal to Cathepsin B (Abcam,ab125067), mouse monoclonal to GAPDH (Abcam, ab8245), SQSTM1/p62 mouse mAb(Cell Signaling Technology,#8588), ATG5 rabbit mAb (Cell Signaling Technology,#12994), ATG12 rabbit mAb (Cell Signaling Technology,#4180), ATG14 rabbit mAb (Cell Signaling Technology,#96752), Erk1/2 rabbit mAb(Cell Signaling Technology,#4695), p-Erk1/2 rabbit mAb (Cell Signaling Technology,#8544), p38 MAPK rabbit mAb (Cell Signaling Technology,#14451), p-p38 rabbit mAb (Cell Signaling Technology,#4631), SAPK/JNK rabbit mAb (Cell Signaling Technology,#9252), Phospho-SAPK/JNK Rabbit mAbp-JNK (Cell Signaling Technology,#4668), were used according to the protocol.

### Cell Culture Conditions

MNNG/HOS and MG-63 human osteosarcoma cell lines and MC3T3-E1 cell line were purchased from the ATCC. MG-63 and MNG/HOS cells were cultured in DMEM (Gibco) with 10% FBS (Biological Industries), and 1% antibiotics (100 U/ml penicillin, 100 μg/ml streptomycin) at 37°C and 5% CO_2_. MC3T3-E1 was cultured in α-MEM with 10% FBS (Biological Industries), and 1% antibiotics (100 U/ml penicillin, 100 μg/ml streptomycin) at 37°C and 5% CO_2_. Treatment reagents were used at the following concentrations: dihydroartemisinin as indicated, 5 mM NAC, 50μM DFO, and 100 μM FAC. DMSO was used as control.

### Cell Viability and Colony Formation Measurement

MG-63 and MNNG/HOS cell viability were measured by CCK-8 assay. 8*10^3^ cells/well were plated in 96-well plates with 200 µl medium. After incubation for overnight, cells were treated with 0~128 μM DHA for 24 h, 48 h, and 72 h. Then, 10% CCK-8 was added to the culture medium and incubated for 2h at 37°C. The absorbance of was measured at 490 nm by using the BioTek microplate reader. For tumor colony formation assays, 1000 cells/dish were plated in 35 mm culture dishes. Then, cells were treated with DHA (10, 20, and 40 μM) or 0.1% DMSO for 24 h. The medium with DHA or DMSO was replaced with fresh medium and colonies were observed after a further 10 days. Then the cells were fixed with 4% paraformaldehyde for 5 min and stained by crystal violet. The photographs of the colonies were taken manually.

### 5-Ethynyl-20-Deoxyuridine (EdU) Incorporation Assay

2*10^5^ osteosarcoma cells per well were seeded in 6-well plates. Cells were treated with DHA at 10, 20, and 40 μM concentrations and 0.1% DMSO as control. After 24 h, 50 μM EdU labeling agent was added per well followed by 8 h incubation and stained according to instructions of test kit. Stained cells were analyzed with an inverted fluorescent microscope under a 20x objective (Leica DMIL, Germany). The fluorescence intensity was analyzed by Image J software.

### AO/EB Staining

5*10^4^ MNNG/HOS and MG-63 cells/well were plated in 24-well plates and treated with DHA in 10, 20, 40 μM concentrations and 0.1% DMSO as control. After 24 h incubation, 0.2% AO, and 0.2% EB solution were added per well for a further 20 min incubation. Stained cells were measured using an inverted fluorescence microscope under 20x objective (Leica DMIL, Germany). The fluorescence intensity was analyzed by Image J software.

### Measurement of Lysosome Stability

2*10^4^ cells were grown in 96-well plates per well and treated with 0 μM, 10 μM, 20 μM, and 40 μM DHA. After 24 h treatment, lysosomes were stained using the Lysosome Staining Kit, which labeled lysosomes from live cells with green fluorescence at Ex/Em=490/525nm. The lysosome staining solution was added to each well and incubated at 37°C, 5% CO2 for 1 h. The cells were fixed with 4% paraformaldehyde for 5 min and stained with DAPI for 5 min. Cells were observed under an Inverted Fluorescent microscope under a 20x objective (Leica DMIL, Leica Microsystems, Germany). Lysosomal Membrane Permeabilization (LMP) was observed using acridine orange (AO) and Lyso-tracker probe. Cells after DHA treatment were stained with AO (5 ug/ml) or Lyso-tracker probe (1:100000) in DMEM at 37°C for 15 min. Stained cells were observed with a confocal laser scanning microscope under a 63x objective (TCS, SP5, Leica Microsystems, Germany).

### ROS Measurement

Cells were seeded in 6-well plates and treated with 0 μM, 10 μM, 20 μM, and 40 μM DHA. DCFH–DA was added to incubated cells for 30 min at 37°C and an excess amount of the probe was removed by washing with DMEM. The ROS production was determined using a flow cytometer (BD FACSCalibur; CA, USA).

### Measurement of Mitochondrial Membrane Potential

JC-1 probe was used to stain the osteosarcoma cells treated with 0, 10, 20, and 40 μM DHA for 24 h. The excess probe was removed by washing with PBS. The level of JC-1 was determined using a flow cytometer (BD FACSCalibur; CA, USA).

### Transmission Electron Microscopy

Cells were cultured in 90 mm dishes and treated with 20 μM DHA for 24 h. After DHA treatment, cells were collected and fixed with 3% glutaraldehyde solution. Then the fixed cells were dehydrated with 30%→50%→70%→80%→90%→95%→100% acetone step by step. The dehydrated cells were embedded with epoxy resin and cut into 50 nm pieces. The samples were stained with uranium acetate, then with lead citrate, at room temperature for 15–20 min, and observed using TEM (HT7700, Hitachi).

### Western Blot Analysis

Cells were seeded in 6-well plates at a density of 2.5*10^5^ per well and treated with DHA or NAC, DFO, FAC with concentration gradients (0 μM, 10 μM, 20 μM, and 40 μM) and time gradients (0 h, 3 h, 6 h,12 h, 24 h, and 48 h). After treatment, cells were lysed using a mammalian lysis buffer with protease inhibitor cocktail (Beyotime, China). The protein was separated by 10%–15% SDS-PAGE and then transferred onto a PVDF membrane. After incubation with the specific primary antibody diluted in the ratio of 1:1000 in TBST solution against the target protein and the species-specific secondary antibody diluted in the ratio of 1:10000 in TBST solution with 5% nonfat milk conjugated to horseradish peroxidase, immunoreactive bands were detected by ECL Plus Western Blotting Detection System (T5200, Tanon). The band densities were measured using Image J software.

### Immunofluorescence Analysis

Cells were seeded in 6-well plates at a density of 2.5*10^5^ per well and treated with DHA for 24 h. Cells were incubated with LC3B or cathepsin B antibody at 4°C overnight and then incubated with FITC-labeled Anti-Rabbit IgG (Beyotime, China) for 50 min at 25 °C. Stained cells were observed using a fluorescence microscope under a 20x objective (Nikon 80i). The fluorescence intensity was analyzed by Image J software.

### Animal Treatment

Male BALB/c-nu mice were obtained at 5 weeks of age and housed in a standard animal laboratory. Animals were cared in accordance with institution guidelines. The animal study was reviewed and approved by the lab of animal experimental ethical inspection, NPU. MNNG/HOS cells were resuspended in PBS with a density of 1*10^7^/ml. Then 200 μl cell suspension was injected subcutaneously per mouse. After tumor formation, mice were divided into three groups, control group, cisplatin group, and 50 mg/kg DHA group according to tumor volume. DHA was suspended in 10% sesame oil + 90% saline. DHA group received an intraperitoneal injection of 100 μl DHA at the dose of 50 mg/kg/d every other day. The control group received an intraperitoneal injection of 100 μl 10% sesame oil + 90% saline every other day, while the cisplatin group was injected with 100 μl cisplatin (6 mg/kg/week, diluted with physiological saline). After 7 days of drug administration, the mice were sacrificed, and the tumors were removed, weighed, and fixed for use in immunohistochemical experiments.

### Measurement of Iron Content

The cells were cultured in a 6-well culture plate, washed with normal saline, dissolved in 65% nitric acid at 70°C for 2 h, and the dried samples were dissolved in 10 ml of 1% nitric acid and 0.1% potassium chloride. The iron content in cells was determined by graphite furnace atomic absorption spectrometry (AAS; Analytik Jena,Germany) and normalized to total protein content. The total iron content in tissues was detected by Prussian Blue staining. The procedure was performed as described in our previous work (Yang et al., 2017).

### Immunohistochemistry Analysis

The tumor tissues were fixed with 4% paraformaldehyde and embedded with paraffin. Embedded tissues were cut into 2 μm slices. Put the section into xylene I 15 min—xylene II 15 min—xylene III 15 min—anhydrous ethanol I 5 min—anhydrous ethanol II 5 min—85% ethanol 5 min—75% ethanol 5 min—distilled water washing. The tissue sections were placed in the repair box filled with citric acid antigen repair buffer (pH6.0) and repaired in the microwave oven. The slices were put into 3% hydrogen peroxide solution and incubated at room temperature in dark for 25 min. The slides were placed in PBS (pH7.4) and shaken and washed for three times on the bleaching shaker for 5 min each time. Then 3% BSA was dripped into the histochemical circle to cover the tissue uniformly, and the tissue was sealed at room temperature for 30 min. Gently shake off the sealing solution, drop PBS on the slice to prepare the first antibody in a certain proportion, and lay the slice flat in the wet box at 4° C for incubation overnight. Put the slide in PBS (pH7.4) and shake it on the bleaching shaker for three times, each time for 5 min. After the slices were slightly dried, the HRP labeled tissues of the corresponding species of the first antibody were dripped into the circles and incubated at room temperature for 50 min. Put the slide in PBS (pH7.4) and shake it on the bleaching shaker for three times, each time for 5min. After the section is slightly dried, add DAB color developing solution in the circle. Control the color developing time under the microscope. The positive color is brown yellow. Wash the section with tap water and stop the color developing. The hematoxylin was re-dyed for about 3 min, washed with tap water, differentiated with hematoxylin differentiation solution for several seconds, washed with tap water, returned to blue with hematoxylin back to blue, washed with running water. Put the slice into 75% alcohol for 5 min—85% alcohol for 5 min—anhydrous alcohol for 15 min—anhydrous alcohol for 5min—xylene for 5 min in in order to dehydrate and make it transparent. Take out the slice from xylene and dry it slightly, and seal it with neutral gum.

### Statistical Analysis

All the experiments were repeated in triplicates. All experimental data are expressed as mean ± SD. All statistically experimental data were assessed by performing a normality test and the data are normally distributed. The significant differences between control and treated groups were determined using the t-test or ANOVA.

## Results

### DHA Inhibits Osteosarcoma Cell Viability, Proliferation, and Induces Cell Death *In Vitro*

**Figure 1:**
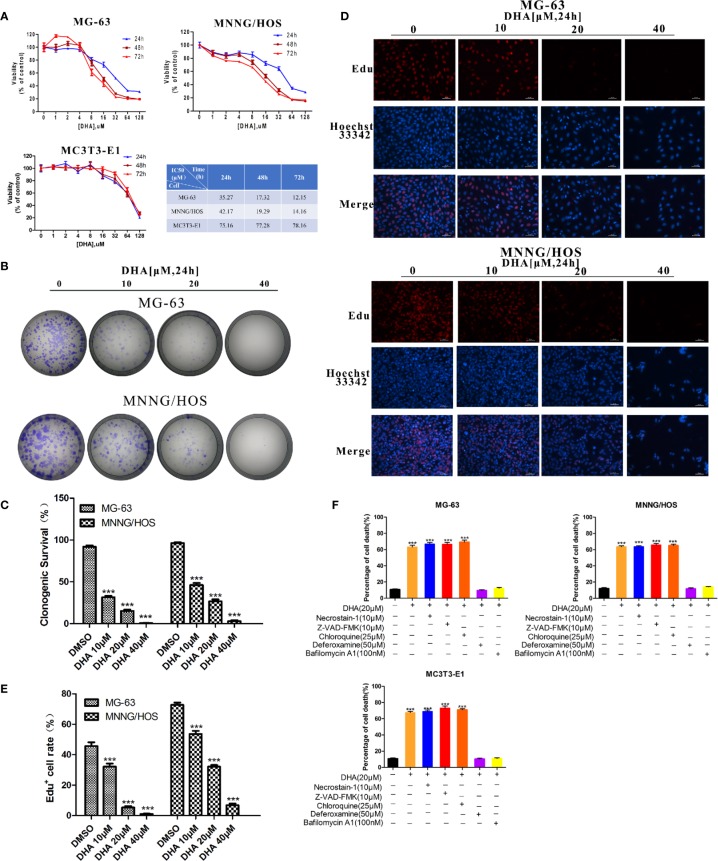
DHA inhibits osteosarcoma cell viability, proliferation and induces cell death *in vitro*. **(A)** Cell viability assays for MG-63, MNNG/HOS and MC3T3-E1 cell lines. Cells were treated with varying concentrations of DHA for 24 h, 48 h, and 72 h, following which cell viability was measured *via* a CCK-8 assay at the indicated time points (n = 5, mean ± SD). **(B, C)** The effect of DHA on the clonogenic ability of MG-63 and MNNG/HOS cells (n = 3). Cells were incubated with 10 μM, 20 μM, and 40 μM of DHA for 24 h. **(D, E)** EdU staining of MG-63 and MNNG/HOS cells. Cells were incubated with 10 μM, 20 μM, and 40 μM of DHA for 24 h and cells were observed using a fluorescence microscope at 20× objective(n = 3). **(F)** MG-63, MNNG/HOS and MC3T3-E1 cells were treated with 20 μM DHA combined with the cell death inhibitors for 24 h and the percentage of cell death was analyzed through cell count (n=5, mean ± SD); ***P < 0.001 versus control. Scale bar = 50 μm.

To observe the effects of DHA on osteosarcoma cell viability we used CCK-8 assay. MG-63, MNNG/HOS, and MC3T3-E1 cells were treated with an increasing concentration of DHA. In **Figure 1A**, CCK-8 analysis show that the DHA reduced MG-63 and MNNG/HOS cell viability in a dose- and time-dependent manner. However, the IC_50_ of DHA in MG-63 and MNNG/HOS cells was significantly lower than that in the osteoblast precursor cell line MC3T3-E1. This indicates that osteosarcoma cells were significantly more sensitive to DHA.

Colony numbers for MG-63 and MNNG/HOS cell lines also decreased with DHA treatment for 24 h (**Figure 1B**). Also, as the concentration of DHA increased, it significantly inhibited colony formation. DHA at 40 μM completely abolished MG-63 and MNNG/HOS colony formation (**Figures 1B, C**). These results suggest that DHA could significantly inhibit the colony forming efficiency of MG-63 and MNNG/HOS.

An EdU incorporation assay was used to further test the effects of DHA on proliferation of MG-63 and MNNG/HOS cells. We observed a significant and dose-dependent reduction in EdU-positive cells in MG-63 and MNNG/HOS cells treated with DHA compared to control (**Figures 1D, E**). Overall, these data indicate that DHA could inhibit the cell viability and proliferation of MG-63 and MNNG/HOS cells.

The acridine orange/ethidium bromide (AO/EB) staining assay was used to observe the effects of DHA on cell death of MG-63 and MNNG/HOS cells. AO stains live cells bright green fluorescence. Ethidium bromide (EB) stains dead cells an orange–red fluorescence. AO/EB staining can therefore be used to detect the ratio of live to dead cells. DHA treatment significantly increase in the number of cells with red fluorescence and decrease in the number of cells with green fluorescence, in a dose-dependent manner (**Figure S1A**). Furthermore, 40μM DHA cannot induce cell death significantly in normal mc3T3 cells (**Figure S1B**). This result suggests that DHA could induce cell death in MG-63 and MNNG/HOS cells and osteosarcoma cells were more susceptible to DHA.

To further validate that DHA induced the type of cell death in MG-63 and MNNG/HOS cells, we exposed DHA treated MG-63, MNNG/HOS, and MC3T3-E1 cells to different cell death inhibitors, including deferoxamine (DFO), an iron chelator, necrostatin-1 (a classic inhibitor of TNF-alpha mediated necroptosis), chloroquine (an inhibitor of autophagy), Z-VAD-FMK (a general caspase inhibitor) and bafilomycin A1(an inhibitor of V-ATPase). In **Figure 1F**, we found that DFO and bafilomycin A1, significantly prevented DHA induced reduced cell death in all three cell lines, while necrostatin-1, chloroquine, and Z-VAD-FMK showed no change compared to the control group (**Figure 1F**). We also noted that the mechanism by which DHA induced cell death in normal osteoblast (MC3T3-E1) is same as that in the osteosarcoma cells (MG-63, MNNG/HOS). These data imply that DHA induced cell death *via* iron and lysosome mediated pathway and is not dependent on the caspase pathway.

### DHA Inhibits Osteosarcoma Growth *In Vivo*

**Figure 2:**
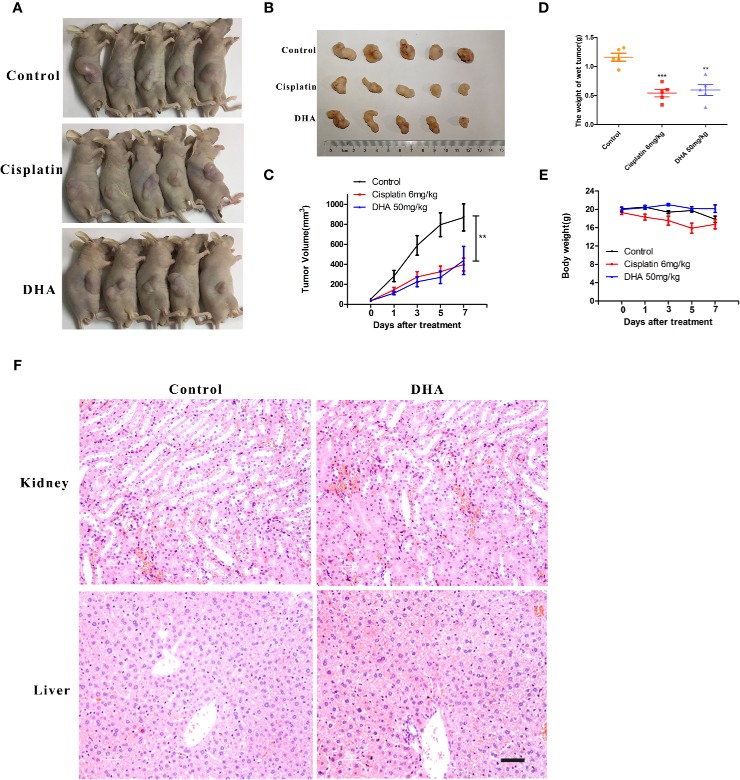
DHA inhibits growth of osteosarcoma *in vivo*. MNNG/HOS cells were injected into BALB/c-nu mice subcutaneously. One week after tumor inoculation, mice were randomly divided into three groups for treatment with either intraperitoneal administration of vehicle, Cisplatin (6 mg/kg/week) or DHA (50 mg/kg/day) for seven days. **(A–C)** Data of tumor volume in mice of three groups (n=5), **(D)** Tumor weight (n=5), **(E)** Measurement of body weight every two days (n=5), **(F)** Organ-related toxicities were assessed with histological H&E staining; **P < 0.01 versus control, ***P < 0.001 versus control. Scale bar = 50 μm.

The *in vivo* effect of DHA on osteosarcoma was measured in tumor- mouse model. In this experiment, cisplatin was used as a positive control drug. We found that 50 mg/kg of DHA significantly inhibited tumor volume and tumor weight after 7 days of drug treatment compared to the control (**Figures 2A–D**). Furthermore, there was no significant difference between the antitumor effect of DHA and cisplatin. Interestingly, we observed no significant decrease in body weight in DHA-treated mice, while the cisplatin-treated mice had a significant decrease in body weight (**Figure 2E**). Hematoxylin and eosin staining of liver and kidney tissue demonstrated that there was no detectable liver and kidney toxicity after DHA treatment (**Figure 2F**). These data suggest that DHA effectively inhibited osteosarcoma growth *in vivo* and prevented adverse effect of weight loss as seen in cisplatin.

### DHA Induces Autophagic Flux Blockage and Mitochondrial Damage *via* ROS Production in Osteosarcoma Cells

**Figure 3:**
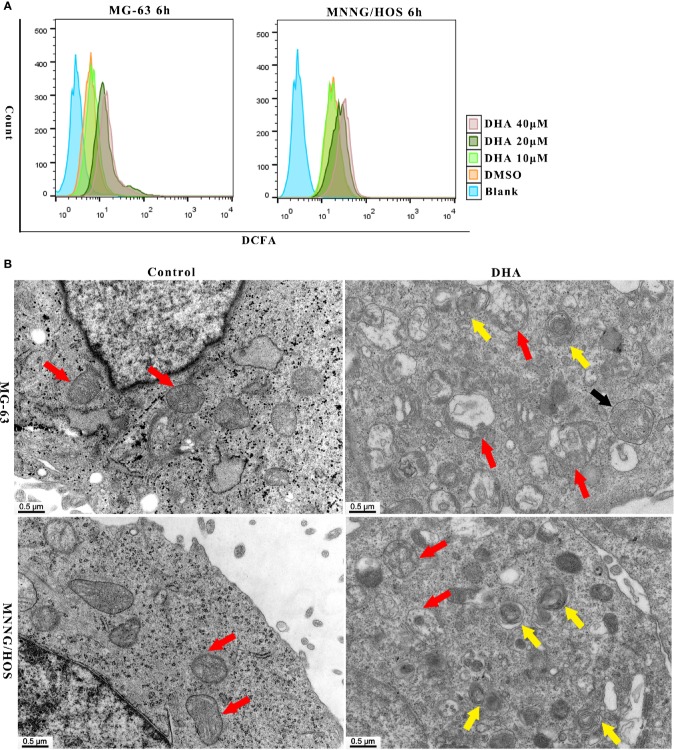
DHA induced ROS generation, mitochondrial damage and autophagosome and autolysosome formation in MG-63 cells and MNNG/HOS cells. **(A)** Analysis of ROS generation in MG-63 and MNNG/HOS treated with 10 μM, 20 μM, 40 μM after 6 h and the cells were analyzed with a flow cytometer (n=3). **(B)** Representative transmission electron micrographs of subcellular structure in control MNNG/HOS and MG-63 cells and 20 μM DHA treated cells for 24 h (n=3).

It is well established that DHA requires iron to cleave its inner endoperoxide bridge to release ROS (Antoine et al., 2014). We hypothesized that iron-rich lysosomes may facilitate the release of ROS by DHA. To quantify ROS production, we used DCFH-DA (2,7-Dichlorodi -hydrofluorescein diacetate) to label cellular ROS that could be detected the by flow cytometry. In **Figure 3A**, we found that ROS production increased in MG-63 cells and MNNG/HOS cells with DHA treated for 6 h. Thus, suggesting that DHA treatment could induce cellular ROS production.

ROS production could be observed in cells just 12 h and 24 h after DHA treatment. (**Figures S2A, B**). The DCFH-DA fluorescence intensity significantly increased after DHA treatment compared to the control group. Thus, ROS production in cells could have positive correlation to the DHA concentration and the duration of DHA treatment.

Previous studies have shown that ROS production could lead to organelle damage (Jiang et al., 2019; Zhang et al., 2019). In order to investigate changes in organelle structure of DHA treated MG-63 and MNNG/HOS cells, transmission electron microscopy (TEM) was used. In **Figure 3B**, mitochondrion were indicated with red arrow, autophagosome was indicated with black arrow and autolysosomes were indicated with yellow arrow. From **Figure 3B**, we could see the intracellular changes, the mitochondria are swollen and damaged, autophagosome and autolysosome are formed in DHA treated cells compared with control cells. In MNNG/HOS cells treated with DHA, we can observe that the autolysosomes were accumulated in cells. Moreover, in the DHA treated cells we found swelling of endoplasmic reticulum. Prior studies have reported that endoplasmic reticulum swelling could be caused by several factors, including oxidative stress (Kim et al., 2019), homocysteine (Wu X. et al., 2019), and dysregulation of calcium homeostasis (Ham et al., 2019). Thus, indicating that the swelling of endoplasmic reticulum could be as a result of oxidative stress caused by ROS generation induced by the DHA in MG-63 cells.

As the mitochondrial membrane potential (MMP) is a major indicator of mitochondrial function, we tested the MMP using the JC-1 probe. After DHA treatment in MG-63 and MNNG/HOS cells for 24 h, the MMP decreased in a dose-dependent manner (**Figures S2C, D**). Overall, the above data indicate that DHA-treatment induced ROS production, mitochondrial damage and autolysosome formation.

**Figure 4:**
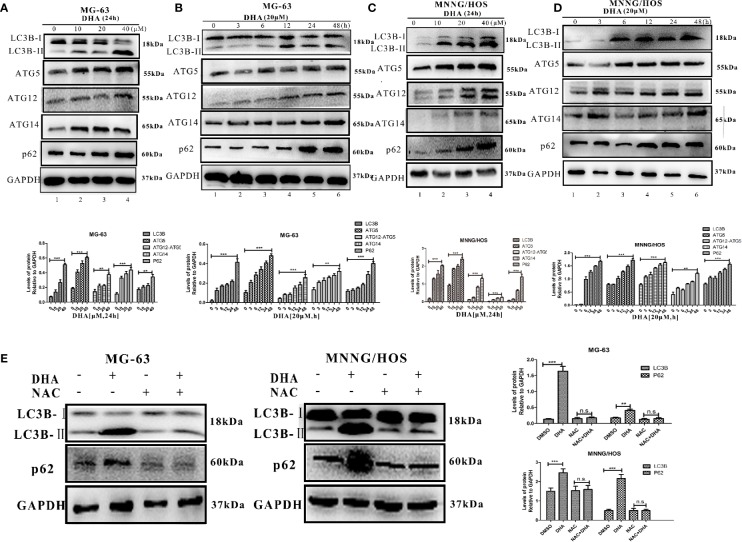
DHA induced autophagic flux blockage *via* ROS production in osteosarcoma. Autophagy-related protein expression was measured by Western blot in **(A)** MG-63 cells treated with 10 μM, 20 μM, and 40 μM DHA for 24 h. (n = 3) **(B)** MG-63 cells treated with 20 μM at various time points (n = 3). **(C)** MNNG/HOS cells treated with 10 μM, 20 μM, and 40 μM DHA for 24 h (n = 3). **(D)** MNNG/HOS cells treated with 20 μM at various time points. (n = 3). **(E)** LC3B and p62 expression were measured in 20 μM DHA treated MG-63 and MNNG/HOS cells for 24 h, with or without 5 mM NAC pre-treatment (n = 3); **P < 0.01 versus control, ***P < 0.001 versus control, n.s., none significance.

Numerous studies have demonstrated that ROS production and mitochondrial damage trigger autophagy in several types of cancer cell (Apel and Hirt, 2004; Park et al., 2019). Thus, we further surmised that DHA induced ROS production could lead to activation of autophagic flux in osteosarcoma cells. To test our hypothesis, we analyzed autophagic flux in MG-63 and MNNG/HOS cells after DHA treatment. LC3B II, an upstream autophagy marker, was significantly increased with DHA treatment in a dose- and time-dependent manner (**Figures 4A–D**). The expression of the autophagy related proteins, ATG5, ATG12-ATG5, and ATG14, were also elevated after DHA treatment in a dose- and time-dependent manner. Furthermore, the expression of p62, a protein which is incorporated into the autophagosome and then degraded by lysosomes, was found to be accumulated in DHA-treated osteosarcoma cells compared to the control. In the MG-63 cells, the LC3 II expression continuously increased, while p62 accumulated as the DHA concentration increased with longer durations (**Figures 4A, B**). Similar results were also observed in MNNG/HOS cells (**Figures 4C, D**).

Immunofluorescence analysis further supports our findings that DHA treatment could increase LC3B expression in osteosarcoma cells (**Figures S3A, B**), further more immunohistochemistry analysis of the osteosarcoma tissue show that DHA treatment promoted LC3B expression *in vivo* (**Figure S3C**). Together, these data indicate that DHA induced upstream autophagic processes but obstructed downstream degradation processes. Thus, the autophagic flux is blocked by DHA treatment in osteosarcoma cells.

It has been reported that the toxicity of DHA is derived from the generation of ROS in the treatment of malaria (Antoine et al., 2014). We predict that DHA induces cell death through ROS generation. In order to test our hypothesis, we pre-treated MG-63 and MNNG/HOS cells with a ROS scavenger, NAC, before DHA treatment, and then performed AO/EB staining. We observed no change in the number of AO positive cells and EB positive cells compared to the control group, (**Figure S4A**). These results suggest that NAC pre-treatment could prevent the cell death induced by DHA in MG-63 and MNNG/HOS cells. Furthermore, analysis of LC3B II and p62 expression indicated that autophagy had not been activated in DHA treated cells with NAC pre-treatment (**Figure 4E**). The JC-1 labeling assay was also used to test the protective effect of NAC on the mitochondrial membrane potential. JC-1 labeling was significantly increased after DHA treatment while the ratio of green/red fluorescence did not change in cells pre-treated with NAC compared to control cells (**Figure S4B**). These data suggest that the mitochondrial membrane potential was maintained in the NAC pre-treatment group. Thereby implying that DHA-induced cell death, mitochondrial membrane potential decline and autophagic flux blockade could be initiated from the ROS production in osteosarcoma cells.

### DHA Activated Autophagy *via* ROS/Erk1/2 Pathway in Osteosarcoma Cells

**Figure 5:**
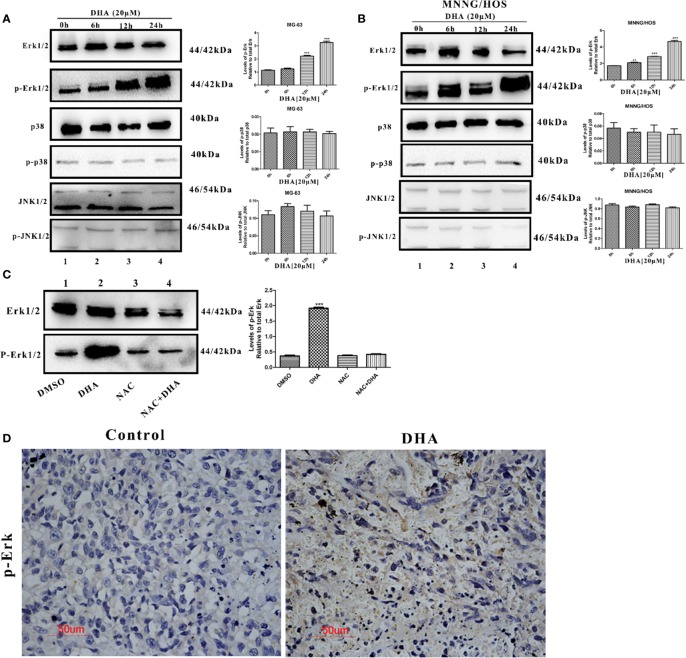
DHA activated autophagy *via* ROS/Erk1/2 pathway in osteosarcoma cells. **(A)** MG-63 cells were treated with 20 µM DHA for 0, 6, 12, 24 h. The expression level of MAPKs proteins were detected by immunoblot analysis (n=3). **(B)** MNNG/HOS cells were treated with 20 µM DHA for 0, 6, 12, 24 h. The expression level of p-Erk1/2, Erk1/2, p-JNK1/2, JNK1/2, phospho-p38, and p38 was detected by immunoblot analysis (n=3). **(C)** Immunoblot analysis of phospho- Erk1/2 and Erk1/2 following NAC pretreatment (5 mM) for 1 h, before 20 μM DHA treatment for 24 h (n=3). **(D)** Immunohistochemistry analysis of the expression levels of phospho-Erk1/2 in osteosarcoma tissue treated with or without DHA (50 mg/kg/day). Representative images are presented. (n = 3); **P < 0.01 versus control, ***P < 0.001 versus control. Scale bar = 50 μm.

Several studies have reported that MAPKs pathways are involved in ROS mediated autophagy. MAPKs pathways consist of three main signaling pathways, including ERK1/2 pathway, JNK1/2 pathway, and p38 pathway (Moldogazieva et al., 2018). We hypothesized that MAPKs pathways could be involved in DHA-induced autophagy. Thus, we measured the expression of key proteins in MAPKs pathways. DHA treated MG-63cells showed a significant increase in the expression of p-Erk1/2, however, no change in the expression of p-p38 and p-JNK was observed (**Figure 5A**). Similarly, p-Erk1/2 expression was increased after DHA treatment in MNNG/HOS cells, while the level of p-p38 and p-JNK remained changed (**Figure 5B**). Furthermore, pre-treatment with NAC significantly abrogated the DHA-induced Erk1/2 phosphorylation, demonstrating that Erk1/2 phosphorylation is a ROS-dependent process (**Figure 5C**). These data indicates that DHA could activate ROS/Erk1/2 pathway in osteosarcoma cells. To further assess the activation of the Erk1/2 pathway *in vivo*, we analyzed the p-Erk1/2 expression in osteosarcoma tissues with or without DHA treatment. In **Figure 5B**, we observed a significant increase in p-Erk1/2 expression in tumor tissue with DHA treatment compared to the control (**Figure 5D**). Our data demonstrated that ROS mediated Erk1/2 pathway activation triggers autophagy after DHA treatment in osteosarcoma.

### Lysosomal Membrane Permeabilization Leads to Autophagic Flux Blockade and Cell Death in DHA Treated Osteosarcoma Cells

We observed that p62 accumulated in DHA treated MG-63 and MNNG/HOS cells (**Figures 4A–D**), indicating that autophagic flux was blocked. DHA relies on iron r to cleave its inner endoperoxide bridge to release ROS. Likewise ROS production is known to induce lysosome dysfunction (Florean et al., 2019; Shaikh et al., 2019). As the lysosome is the major organelle containing abundant iron and is also implicated in autophagosomes degradation (Towers and Thorburn, 2017), we assumed that lysosome could be play a role in the DHA induced autophagic flux blockage and cell death. We detected lysosome stability after DHA treatment using a fluorescent lysosome staining kit. Lysogreen probe stains acidic compartments in live cells with green fluorescence indicating the acid lysosomes. In **Figure S5A**, we observed that the DHA treated MG-63 and MNNG/HOS cells had reduction in green fluorescence signal with increase in DHA concentration. These data demonstrate that DHA could be responsible for change in the lysosomal pH.

**Figure 6:**
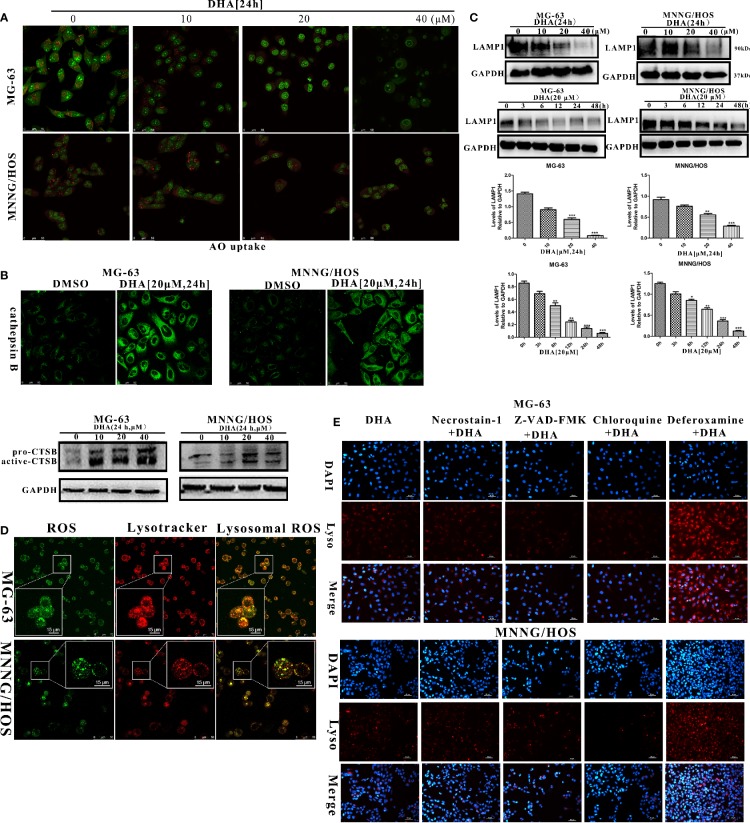
DHA induced lysosomal membrane permeabilization *via* lysosomal ROS production. **(A)** AO uptake analysis of MG-63 and MNNG/HOS cells. Cells were treated with 10 μM, 20 μM, and 40 μM DHA for 24 h and cells were observed using a laser confocal microscope (n = 3). **(B)** Immunofluorescence analysis and western blotting analysis of cathepsin B expression in MG-63 and MNNG/HOS cells treated with or without 20 μM DHA treatment for 24 h. **(C)** LAMP1 expression in MG-63 and MNNG/HOS cells was assessed by immunoblotting, after concentration- and time- dependent DHA treatment. LAMP1 expression were analyzed after 0, 10, 20, and 40 μM DHA treatment for 24 h. **(D)** Lysosome and ROS co-localization analysis of MG-63 and MNNG/HOS cells treated with 20 μM DHA for 3 h. **(E)** LMP analysis of MG-63 and MNNG/HOS cells treated with 20 μM DHA for 24 h combined with Necrostain-1(10 μM), Z-VAD-FMK (10 μM), Chloroquine (25 μM), Deferoxamine (50 μM). *P < 0.05 versus control, **P < 0.01 versus control, ***P < 0.001 versus control. Scale bar = 50 μm.

It has been reported that, lysosomal membrane permeabilization (LMP) is the main reason for the rise of lysosomal pH (De Milito et al., 2007; Gavini et al., 2019). Thus, we further analyzed the LMP stability in DHA treated osteosarcoma cells using Acridine orange (AO) staining and measurement of the release of cathepsin B into the cytosol as described in earlier studies (Li P. et al., 2016; Repnik et al., 2016). At 10 μM DHA treatment, no significant change or reduction in orange fluorescence was observed compared to the control (**Figure 6A**). However, when treated with 20 μM and 40 μM DHA, the number of orange dots was significantly reduced in MG-63 and MNNG/HOS cells. The AO staining assay demonstrated that the DHA at higher dose could induce lysosome membrane permeabilization in MG-63 and MNNG/HOS cells. In **Figure 6B**, the immunofluorescence and western blotting analysis of cathepsin B showed that DHA induced the release of cathepsin B into the cytosol. From **Figure S5A** and **Figures 6A, B**, we can observe that DHA induces lysosome dysfunction and lysosome membrane permeabilization (LMP).

Lysosome-associated membrane glycoprotein 1 (LAMP1) is a key protein which maintains low lysosomal pH and lysosome membrane integrity (Eskelinen, 2006; Alessandrini et al., 2017). In order to analyze lysosomal function, we measured the LAMP1 expression in the osteosarcoma cells with DHA treatment at different time points and concentrations. We found that the LAMP1 expression was significantly decreased on treatment with 20 μM and 40 μM DHA (**Figure 6C**). Moreover, we observed a reduction in LAMP1 expression as early as 3 h after DHA 20 μM treatment. Comparatively, from **Figures 4A–D**, p62 accumulation is seen at 12 h after DHA treatment. These results suggest that LMP precedes blockade of autophagic flux. LMP is induced by multiple factors including reactive oxygen species, lysosomotropic compounds with detergent activity, as well as some endogenous cell death effectors such as Bax (Wang et al., 2018).

We had earlier that in the DHA treated osteosarcoma cells ROS production increased and MMP decrease. To investigate relationship between ROS production, MMP, and LMP, we performed lysosome and ROS co-localization analysis using ROS-Tracker and Lyso-Tracker. In **Figure 6D**, we observed that lysosomal ROS generated with 20μM DHA for 3h. From **Figures S5B, C**, we observed that the MMP decay from 12 h with 20 μM DHA treatment, while the LMP was triggered from 3 h with 20μM DHA treatment. These data further demonstrates that in the chain of events bought about by DHA, lysosomal ROS production precedes LMP instability, followed by MMP instability.

LMP has been shown to be implicated in different types of cell death. In order to analyze the LMP backed type of cell death, LMP stability was observed in DHA treated cells exposed to various cell death inhibitors, including Deferoxamine (DFO), necrostatin-1, chloroquine, and Z-VAD-FMK. From **Figure 6E**, we found that only DFO could prevent the LMP instability, while Z-VAD-FMK, Necrostatin-1, and chloroquine failed to protect LMP stability in DHA treated osteosarcoma cells. These data further illustrate that DHA-induced cell death *via* iron-dependent lysosomal ROS production and LMP instability that lead to the blockade autophagic flux in osteosarcoma cells.

### Iron Is a Necessary Element to Promote the Antitumor Properties of DHA

**Figure 7:**
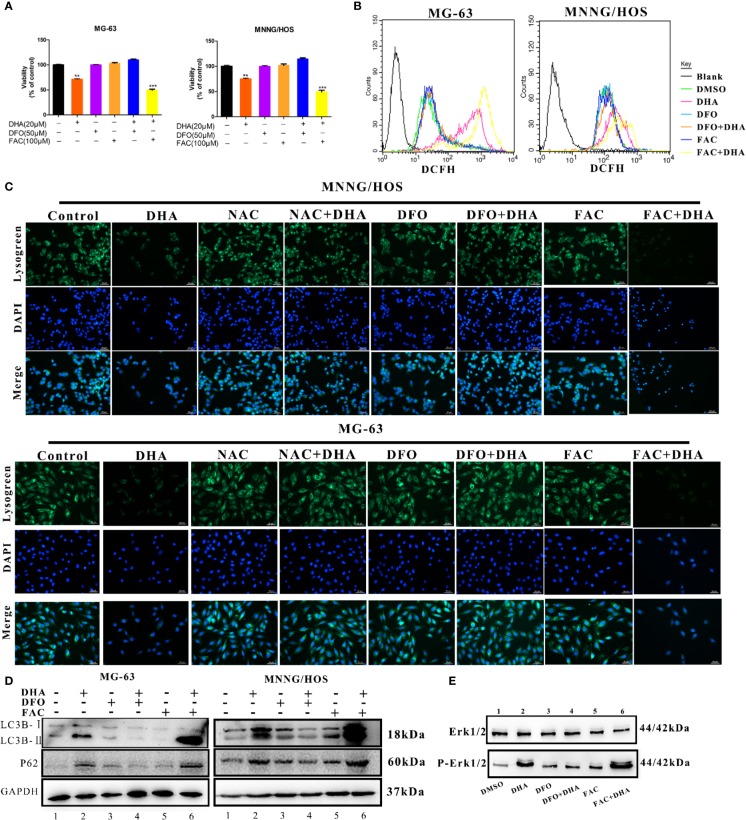
Iron promotes LMP and autophagic blockage *via* ROS production in DHA treated osteosarcoma cells. **(A)** Cell viability assay in MG-63 and MNNG/HOS. Cells were treated with varying concentrations of DHA for 24 h with or without pre-treatment with 50 μM DFO and 100 μM FAC, after which cell viability was measured *via* a CCK-8 assay at the indicated time points (n = 5, mean ± SD). **(B)** ROS generation analysis of MG-63 and MNNG/HOS cells treated with 20 μM DHA for 24 h, with or without pre-treatment with 50 μM DFO and 100 μM FAC. **(C)** Lysogreen staining of 20 μM DHA treated MG-63 cells for 24 h, with or without pre-treatment with 5 mM NAC, 50 μM DFO, or 100 μM FAC. **(D)** LC3B and p62 expression were measured in MG-63 cells and MNNG/HOS cells treated with 20 μM DHA for 24 h combined with or without 50 μM DFO and 100 μM FAC pretreatment. **(E)** Cells were pretreated with DFO (50 μM) and FAC (100 μM) for 24 h followed by DHA treatment. Following the treatment, immunoblot analysis of phospho- Erk1/2 and Erk1/2 was performed. **P < 0.01 versus control, ***P < 0.001 versus control. Scale bar = 50 μm.

DHA requires iron to produce ROS. To directly assess whether iron is a necessary element for the antitumor effects of DHA, MG-63, and MNNG/HOS cells were pre-treated with either the iron chelator deferoxamine (DFO), or the iron supplement ferric ammonium citrate (FAC), at various concentrations. Cell viability was assessed by CCK-8 assay. DFO treatment reduced cell death and increased cell viability, whereas FAC treatment increased cell death and reduced cell viability (**Figure 7A**). Therefore, DFO treatment was able to significantly inhibit the antitumor effect of DHA, while FAC promoted the antitumor effect of DHA in MG-63 and MNNG/HOS cells. We next assessed whether iron affected ROS production in osteosarcoma cells. ROS production was significantly increased in DHA-treated cells with FAC pre-treatment compared to only DHA-treated cells. Compared to control cells, DFO pre-treatment did not significantly alter ROS generation following DHA treatment (**Figure 7B**). This result indicated that DHA-induced ROS generation were iron dependent processes.

**Figure 8:**
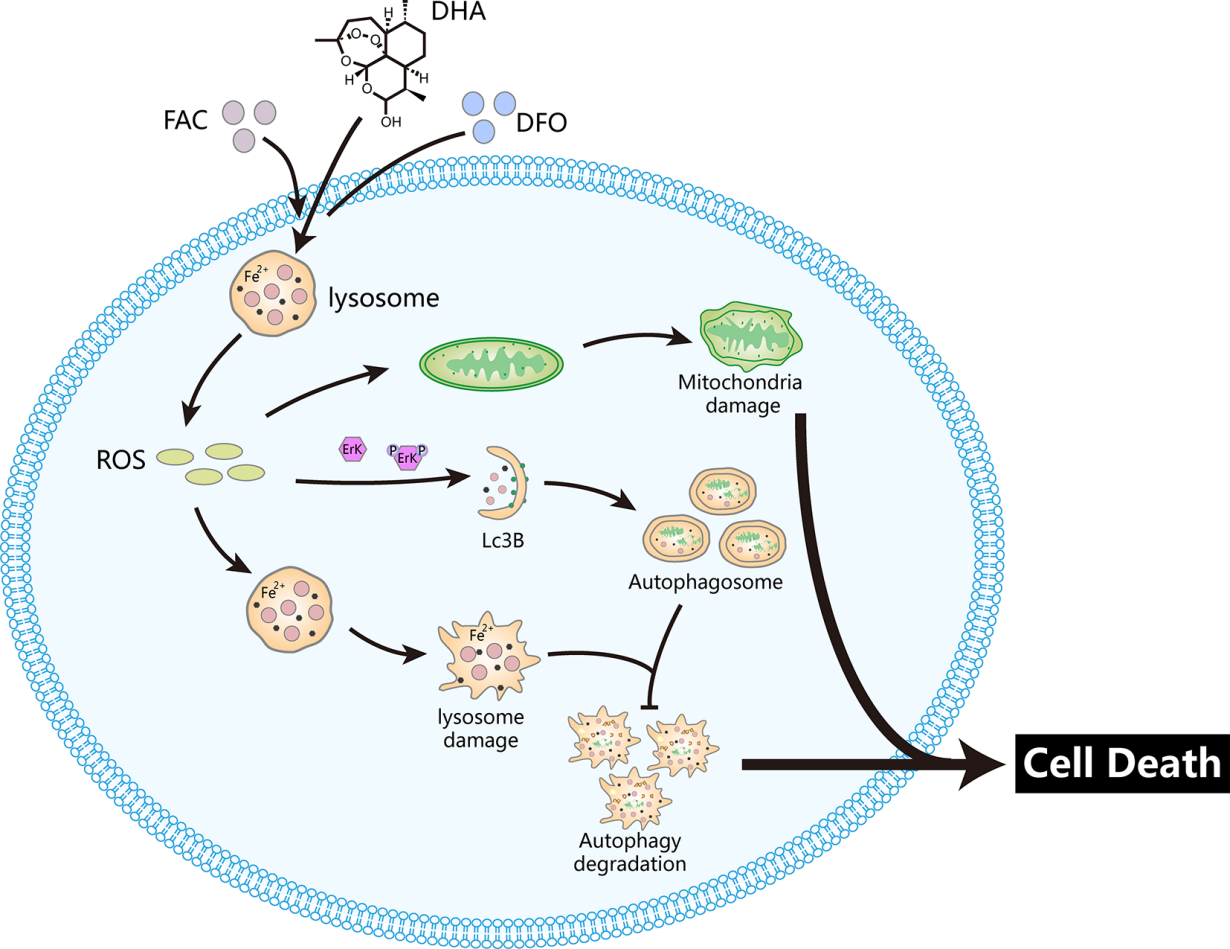
DHA releases ROS via the Fenton reaction in lysosomes, which damages lysosomes and mitochondria. In parallel, ROS production triggers upstream autophagy processes via activation of the Erk1/2 pathway. Damaged lysosomes prevent autophagic degradation. These processes induce osteosarcoma cell death. The iron chelator DFO prevents DHA-induced cell death while the iron supplement FAC promotes DHA-induced cell death.

To further study the effects of iron on lysosomes, we utilized a lysosome staining assay. Lysogreen staining showed DHA-induced lysosome rupture, while NAC and DFO pretreatment protected cells from DHA-induced lysosomal damage. However, FAC pre-treatment promoted DHA-induced lysosome dysfunction (**Figure 7C**). The expression of LC3B II was significantly increased, while p62 was significantly accumulated in DHA-treated cells with FAC pre-treatment compared to DHA-treated cells without FAC pre-treatment. DFO pre-treatment prevented this increase in LC3B II and p62 expression compared to cells treated with DHA alone (**Figure 7D**). This result indicated that FAC promoted autophagic flux blockade on DHA treatment, while DFO prevented the DHA-induced autophagy defects, compared to control cells.

To find the role of iron in DHA-induced ROS/Erk1/2 activation, DFO and FAC were used to pre-treat cells followed by DHA treatment. We found that pretreatment with DFO effectively prevented an increase in p-Erk1/2. Interestingly, DHA-treated cells with FAC pre-treatment showed an increase in p-Erk1/2 expression compared to cells treated with DHA only (**Figure 7E**). Overall, our data shows that DHA-induced ROS production, Erk1/2 activation, and antitumor effects are associated with iron in osteosarcoma cells. Overall, these data demonstrated that iron is a necessary element for DHA antitumor effects, while increasing cellular iron by FAC promotes DHA antitumor activity. Therefore, cellular iron is a potential adjuvant for DHA antitumor treatments.

### High Iron Content in Osteosarcoma Promotes DHA Anti-Osteosarcoma Activity

From this study we uncovered that the iron supplement, FAC, promoted the anticancer effects of DHA and that the Osteosarcoma cells were more sensitive to DHA treatment compared to non-cancerous osteoblast cells. Thus, we hypothesize that cancerous cells more iron-phillic and have increased cellular iron content. In order to test this hypothesis, we analyzed the cellular iron content of non-cancerous osteoblast MC3T3-E1 cells and the osteosarcoma cell lines MG-63, MNNG/HOS, U2OS, and 143B. The data imply that the iron content in osteosarcoma cells was significantly higher than in non-cancerous osteoblast cells. (**Figure S6A**) We also tested the iron content in osteosarcoma tissue, tibia tissue and femur tissue of mice bearing osteosarcoma by Prussian blue staining. Osteosarcoma tissue had a higher level of iron content compared to non-cancerous tissues (**Figure S6B**), which could be propitious to anti-osteosarcoma effects of DHA in a clinical setting. Increased levels of cellular iron in cancerous cells are not only needed to satisfy tumor proliferation, but also catalyze the Fenton reaction to produce reactive oxygen species (ROS), which makes cancer cells extremely vulnerable to the oxidation-reduction equilibrium (Raza et al., 2014). Furthermore, FAC inhibited normal cells cell viability at 640 μM, which is much higher than the concentration we used when combined with DHA. (**Figure S6C**) This characteristic of osteosarcoma could potentially be taken advantage of to develop more targeted anti-cancer drugs. Furthermore, iron supplementation could be explored as a potential adjuvant for DHA.

## Discussion

DHA is a derivative of artemisinin that was identified in the Chinese medicinal herb *Artemisia annua* L., by Youyou Tu. It has been used in the treatment of malaria for several years (Tu, 2016). Previous studies have shown that DHA induces apoptosis, autophagy and ferroptosis of cancer cells *via* caspase activation, cytochrome c release, Mcl-1 down-regulation, MEK/ERK inactivation, JNK/NF-κB pathway activation, AKT-mTOR pathway suppression, and autophagy-dependent degradation of ferritin (Gao et al., 2011; Liu et al., 2018; Wu L. et al., 2019; Zou et al., 2019). In this study, we found that DHA inhibited osteosarcoma proliferation *in vivo* and *in vitro* and induced osteosarcoma cell apoptosis. DHA relies on cellular iron to catalyze the Fenton reaction to generate ROS in osteosarcoma cells. Pre-treatment with the iron chelator DFO abolished DHA-induced ROS production in osteosarcoma cells, while iron supplementation with FAC pre-treatment promoted DHA-induced ROS production. DHA induced ROS generation and Erk1/2 pathway activation, trigging lysosome membrane permeation, and mitochondrial membrane potential decline, which activated upstream autophagic processes. However, p62 accumulated in DHA-treated osteosarcoma cells, indicating that autophagic degradation was blocked. It has been reported that the accumulation of p62 leads to cell apoptosis (Islam et al., 2018). In this study, p62 accumulation occurs after lysosome damage. Thus, p62 may accumulate due to lysosomal dysfunction, which could bring about cell apoptosis after DHA treatment. The ROS scavenger NAC significantly rescued DHA-induced cell death. Thus, we conclude that iron-mediated ROS generation is the major mechanism of DHA toxicity in osteosarcoma.

Osteosarcoma is a common malignant bone tumor in adolescents or children under 20 years of age (Isakoff et al., 2015). However, the clinical treatment of osteosarcoma still lacks efficient targeted drugs. Our laboratory has worked extensively on bone research, and our previous studies have validated that iron metabolism is closely related to bone health (Jia et al., 2014; Zhang et al., 2014; Yang et al., 2017; Yang et al., 2018). Furthermore, other studies have demonstrated that osteosarcoma has a high cellular iron level to support tumor proliferation (Dvořák et al., 2012; Caro et al., 2013). Given that the iron-dependent characteristics of osteosarcoma, we believe that drugs targeting iron may have a high potential to treat osteosarcoma. DHA contains an endoperoxide bridge which is dependent on iron to catalyze the Fenton reaction to release ROS. Importantly, preliminary *in vivo* studies indicate that DHA has therapeutic potential for cancer treatment, and clinical studies have already shown an excellent safety record in malaria treatment, which makes DHA an optimal choice as an effective therapeutic agent for osteosarcoma treatment.

Iron is an essential trace element to support life. Iron metabolism is tightly controlled in humans and maintained by a relative balance between iron absorption and excretion (Rishi et al., 2018). Numerous diseases can lead to abnormal iron metabolism, and abnormal iron metabolism can also cause various diseases (Conrad and Umbreit, 2000). Iron participates in the synthesis of tumor DNA, enzymes, and other biological processes, but could also make tumor cells extremely vulnerable to perturbations in redox balance (Conrad and Umbreit, 2000; Toyokuni, 2019). Iron deprivation and iron overload both have been shown to induce cell death. Li et al. found that a novel iron chelator, Dp44mT, could suppress osteosarcoma proliferation, invasion, and migration *via* ROS production and cell cycle arrest in S phase (Li Y. et al., 2016). Recent studies have found that the inhibition of antioxidant enzymes could induce cancer cell ferroptosis, a new type of cell death triggered by iron-mediated ROS accumulation (Dixon et al., 2012). Thus, the increased cellular iron characteristics of tumor cells provides an efficient base for discovery of effective targeted drugs. Such a high iron level in tumor cells can be exploited to destroy the redox balance of tumor cells and induce cell death. Our study has indicated that increasing cellular iron content can significantly promote the anti-osteosarcoma function of DHA, and further, suggesting that a variety of iron supplements could be developed as suitable adjuvants for DHA in osteosarcoma treatment, potentially giving a novel therapy in the treatment of osteosarcoma tumors.

Lysosomes are attractive targets in the search for anticancer drugs owing to their vital functions and characteristics in cancer cell lines. Specifically, as lysosomes contain iron pools that are the leading site for the Fenton reaction, they must be able to withstand oxidative stress (Dielschneider et al., 2017). Lysosome associated membrane protein-1(LAMP-1) is a major protein component of the lysosome membrane. It has been reported that LAMP-1 is involved in the interaction and fusion of the lysosomes with themselves as well as with other cell components, including endosomes, phagosomes, and the plasma membrane (Eskelinen, 2006). Jiao et al. found that autophagosome-lysosome fusion can be blocked by decreasing the expression of LAMP1 (Jiao et al., 2018). Tingting C et al. found that the upregulation of LAMP1 could accelerate autophagosome formation and degradation in cervical cancer cells (Tingting et al., 2019). Sarkar C et al. reported that the LAMP1 decreased in the lysosomal membrane permeabilization process, which inhibited autophagy flux *in vivo* (Sarkar et al., 2019).

In this manuscript, we found that DHA induced the lysosome membrane permeabilization and decreased the expression of LAMP1. This process may involve the inhibition of autophagy degradation in DHA treated cells. ROS produced by the Fenton reaction has the capacity to cause lysosomal rupture, known as lysosomal membrane permeability (LMP). LMP could then induce mitochondrial damage and autophagy activation (Sironi et al., 2019). Autophagy activation is often considered as a self-rescue behavior of cancer cells, but LMP can block the autophagic degradation process, which could also contribute to cell death. Besides, lysosomal rupture releases lysosomal cathepsins into the cytoplasm, causing degradation of macromolecules and cellular structures (Dielschneider et al., 2017). Lysosomal dysfunction mediates various types of cell death, making lysosomes a potential target for cancer therapy. In our study, iron-rich lysosomes are a site of action for DHA. Based on this result, we can now design drugs to target the iron-rich lysosomal environment.

In ROS-induced autophagy, MAPKs like Erk1/2, JNK1/2, and p38 function as downstream signaling proteins (Liang et al., 2019). In our study, DHA did not affect JNK1/2 or p38 expression, but individually activated Erk1/2. It has been reported that Erk1/2 can regulate multiple signaling pathways and affect tumor development. Among them, autophagy induced by the ROS-/Erk1/2 signaling pathway could induce death in numerous types of tumor cells (Cook et al., 2017; Roskoski, 2019). In our study, we reveal that Erk1/2 could be activated by DHA-induced ROS generation, which triggered cancer cell autophagy. which is also consistent with previous studies. Studies have also shown that the ROS/Erk1/2 pathway could induce other types of cell death. This research indicates that the ROS/Erk1/2 signaling pathway is an important pathway for tumor research and an important target for the discovery of antitumor drugs.

Overall, we propose a potential mechanism for DHA inhibition of osteosarcoma (**Figure 8**). DHA required iron to produce ROS and superoxide, which damaged lysosomes, and activated the ROS/Erk1/2 pathway. Activation of the ROS/Erk1/2 pathway induced autophagy, while damaged lysosomes obstructed autophagic flux, which ultimately lead to cell death in DHA-treated osteosarcoma cells. We demonstrated for the first time that iron is an important adjuvant for the anti-osteosarcoma effects of DHA. This study provides a new perspective on the exploitation of the physiological characteristics of tumor cells to develop targeted drugs.

## Data Availability Statement

The datasets generated for this study are available on request to the corresponding author.

## Ethics Statement

The animal study was reviewed and approved by The lab of animal experimental ethical inspection, NPU.

## Author Contributions

YiS and BZ conceived of the study, performed the investigation, analyzed the results, and wrote the manuscript. PS and SB helped to revise the manuscript. XW, YiS, and XL carried out the cell culture assays. YX, LX, ZW, ZY, and GZ participated in the design of the study. All authors read and approved the final manuscript.

## Funding

This work was supported by the Science and Technology Planning Project of Shenzhen of China (JCYJ20170412140904406), and the National Basic Research Program of China (51777171).

## Conflict of Interest

The authors declare that the research was conducted in the absence of any commercial or financial relationships that could be construed as a potential conflict of interest.

## Supplementary Material

Figure S1 DHA induced osteosarcoma cell apoptosis. **(A)** AO/EB staining of MG-63 and MNNG/HOS cells. Cells were treated with 10μM, 20μM and 40μM DHA for 48h and cells were observed using a fluorescence microscope (n = 3). **(B)** MC3T3-E1 cells were treated with DHA for 24 h and the percentage of cell death was analyzed through cell count.(n=5, mean ± SD); *P < 0.05 versus control, **P < 0.01 versus control, ***P < 0.001 versus control. Scale bar = 50μm. Scale bar = 50μm.

Figure S2 DHA induced ROS production and decreasing of mitochondrial membrane potential in MG-63 and MNNG/HOS cells. **(A)** ROS generation in MG-63 and MNNG/HOS treated with 10μM, 20μM and 40μM DHA after 12h was measured with DCFA probe and assessed by flow cytometry. **(B)** ROS generation in MG-63 and MNNG/HOS treated with 10μM, 20μM and 40μM DHA after 24h was measured with DCFA probe and assessed by flow cytometry. **(C, D)** Measurement of the mitochondrial membrane potential with JC-1 fluorescent probe and flow cytometry. Cells were treated with 10μM, 20μM and 40μM DHA for 24h.

Figure S3 DHA induces LC3B expression in osteosarcoma cells and tissues. **(A, B)** Immunofluorescence analysis of LC3B expression in MG-63 and MNNG/HOS cells treated with or without 20μM DHA treatment for 24h. **(C)** The expression level of LC3B in osteosarcoma tissues treated with 50mg/kg DHA for 7 days was examined by immunohistochemistry. H&E staining was used to measure the histology. Representative images are presented; *P < 0.05 versus control, **P < 0.01 versus control, ***P < 0.001 versus control. Scale bar = 50μm.

Figure S4 NAC protects osteosarcoma cells from cell death and mitochondrial membrane potential decrease induced by DHA. AO/EB staining of 20μM DHA-treated MG-63 **(A)** and MNNG/HOS **(B)** cells, with or without 5mM NAC pre-treatment for 24h. **(B)** Measurement of mitochondrial membrane potential with JC-1 fluorescent probe and flow cytometry following 20μM DHA treatment for 24h in MG-63 cells, with or without 5mM NAC pre-treatment. *P < 0.05 versus control, **P < 0.01 versus control, ***P < 0.001 versus control. Scale bar = 50μm.

Figure S5 DHA induced LMP and MMP decade. **(A)** Lysogreen staining of MG-63 and MNNG/HOS cells. Cells were treated with 10μM, 20μM and 40μM DHA for 24h and cells were observed using a fluorescence microscope (n = 3). **(B)** Lysogreen staining of MG-63 cells and MNNG/HOS cells with 20μM DHA treatment at 0h, 3h, 6h, 12h, 24h were analyzed by flow cytometry. (n=3) **(C)** JC-1 staining of MG-63 cells and MNNG/HOS cells with 20μM DHA treatment at 0h, 3h, 6h, 12h, 24h were analyzed by flow cytometry. (n=3) Cells were observed with 20× objective. Scale bar = 50μm.

Figure S6 High iron content in osteosarcoma promotes the anti-osteosarcoma properties of DHA. **(A)** Iron content in non-cancerous osteoblast and osteosarcoma cells. **(B)** Iron content in mouse tibia, mouse femur and osteosarcoma tissue. **(C)** Cell viability assays for MC3T3-E1 cell lines treated with FAC at different concentrations. (n=5) *P < 0.05 versus control, **P < 0.01 versus control, ***P < 0.001 versus control.

Figures S7–S11 Original image files of the blots included in the article Figures.

## Abbreviations

AO, acridine orange; DFO, deferoxamine; DHA dihydroartemisinin; FAC, ferric ammonium citrate; LMP, lysosomal membrane permeabilization; NAC, N-acetyl-L-cysteine; MMP, mitochondrial membrane potential; ROS, reactive oxygen species.
