# Supplementary figures and images for: Iron Promotes Dihydroartemisinin Cytotoxicity via ROS Production and Blockade of Autophagic Flux via Lysosomal Damage in Osteosarcoma
Source: Front Pharmacol. 2020 May 5;11:444. doi: 10.3389/fphar.2020.00444 (PMC7214747; doi:10.3389/fphar.2020.00444)

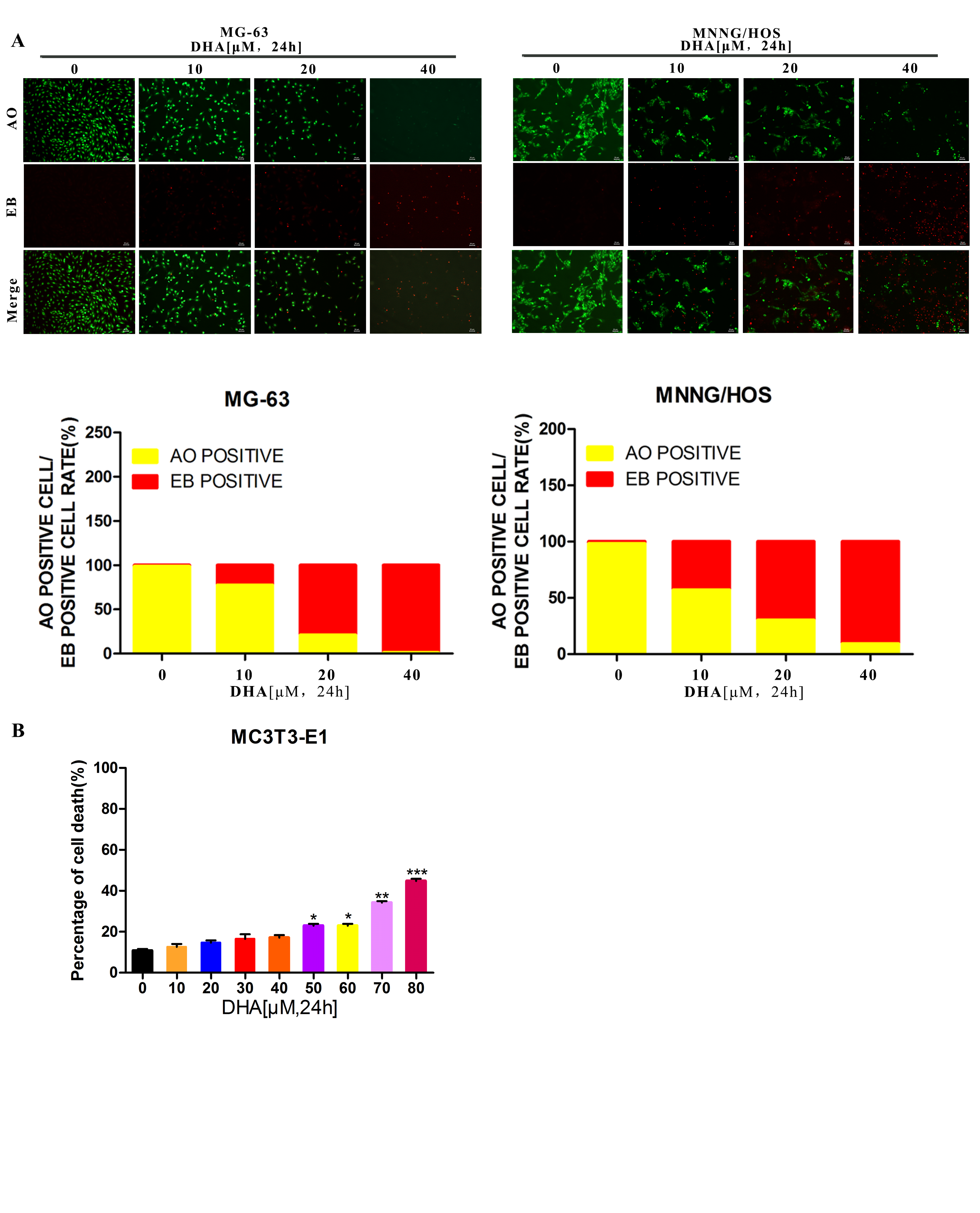

Supplement: Figure S1 — DHA induced osteosarcoma cell apoptosis. (A) AO/EB staining of MG-63 and MNNG/HOS cells. Cells were treated with 10μM, 20μM and 40μM DHA for 48h and cells were observed using a fluorescence microscope (n = 3). (B) MC3T3-E1 cells were treated with DHA for 24 h and the percentage of cell death was analyzed through cell count.(n=5, mean ± SD); *P < 0.05 versus control, **P < 0.01 versus control, ***P < 0.001 versus control. Scale bar = 50μm. Scale bar = 50μm. [file Image_1.tif]

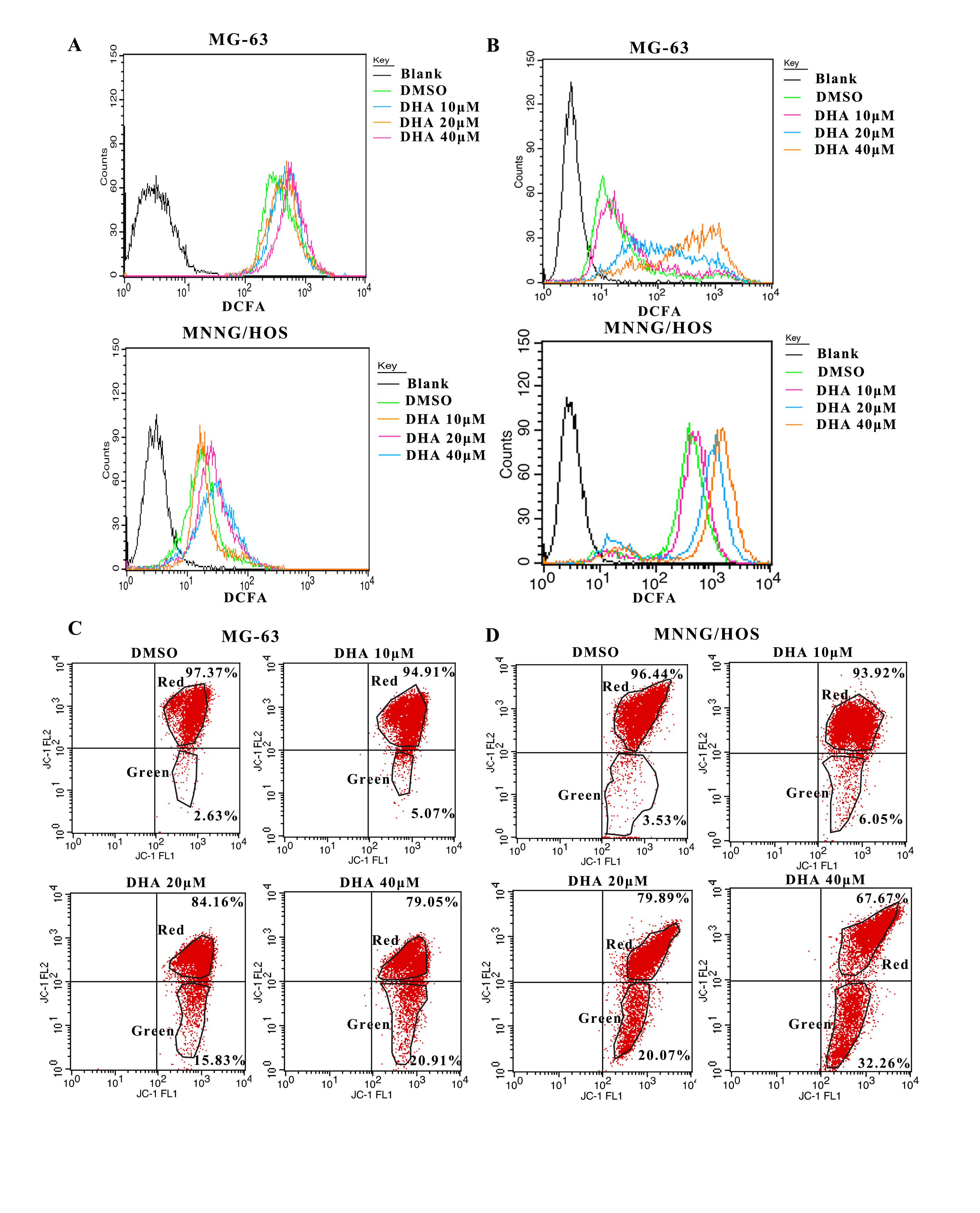

Supplement: Figure S2 — DHA induced ROS production and decreasing of mitochondrial membrane potential in MG-63 and MNNG/HOS cells. (A) ROS generation in MG-63 and MNNG/HOS treated with 10μM, 20μM and 40μM DHA after 12h was measured with DCFA probe and assessed by flow cytometry. (B) ROS generation in MG-63 and MNNG/HOS treated with 10μM, 20μM and 40μM DHA after 24h was measured with DCFA probe and assessed by flow cytometry. (C, D) Measurement of the mitochondrial membrane potential with JC-1 fluorescent probe and flow cytometry. Cells were treated with 10μM, 20μM and 40μM DHA for 24h. [file Image_2.tif]

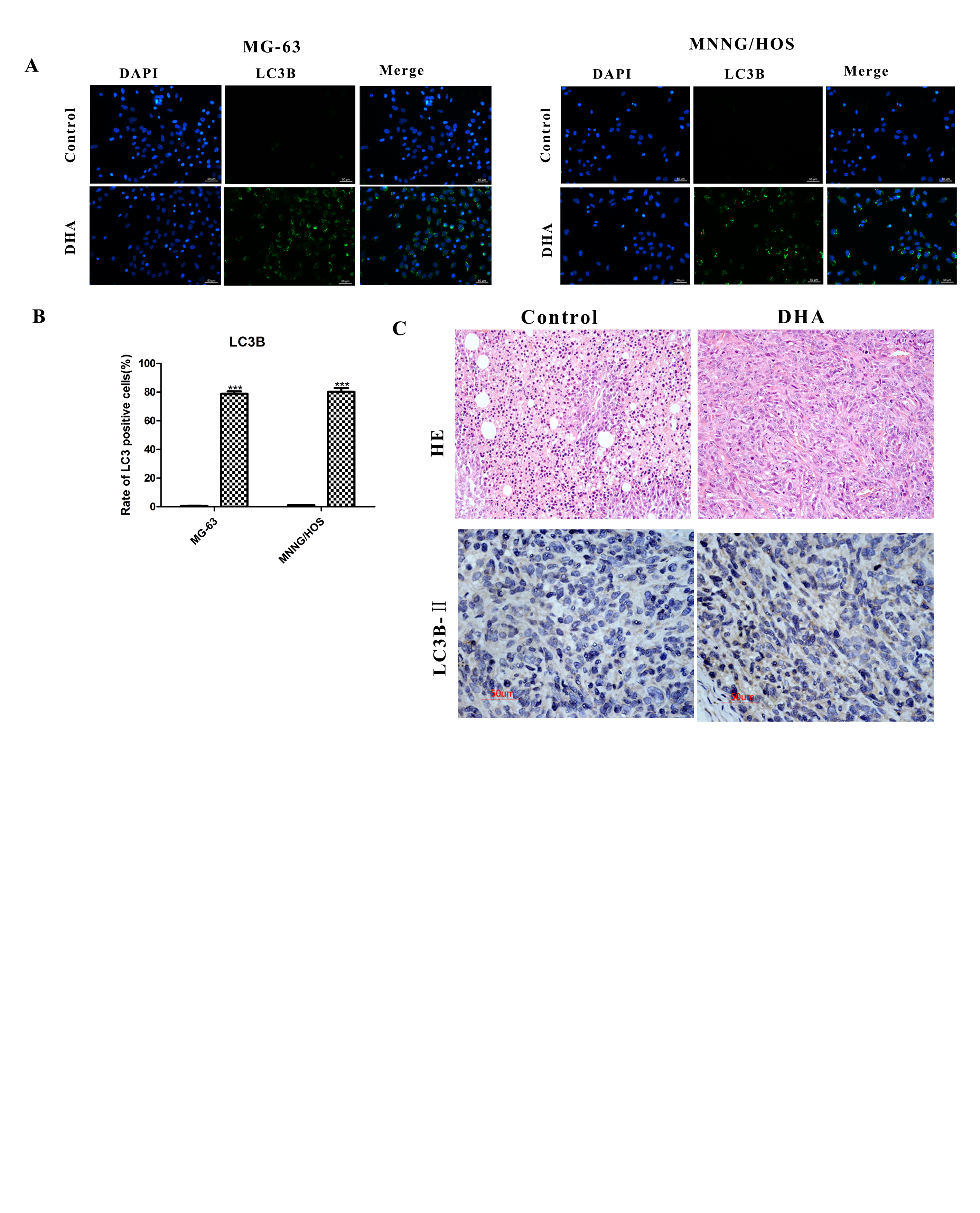

Supplement: Figure S3 — DHA induces LC3B expression in osteosarcoma cells and tissues. (A, B) Immunofluorescence analysis of LC3B expression in MG-63 and MNNG/HOS cells treated with or without 20μM DHA treatment for 24h. (C) The expression level of LC3B in osteosarcoma tissues treated with 50mg/kg DHA for 7 days was examined by immunohistochemistry. H&E staining was used to measure the histology. Representative images are presented; *P < 0.05 versus control, **P < 0.01 versus control, ***P < 0.001 versus control. Scale bar = 50μm. [file Image_3.tif]

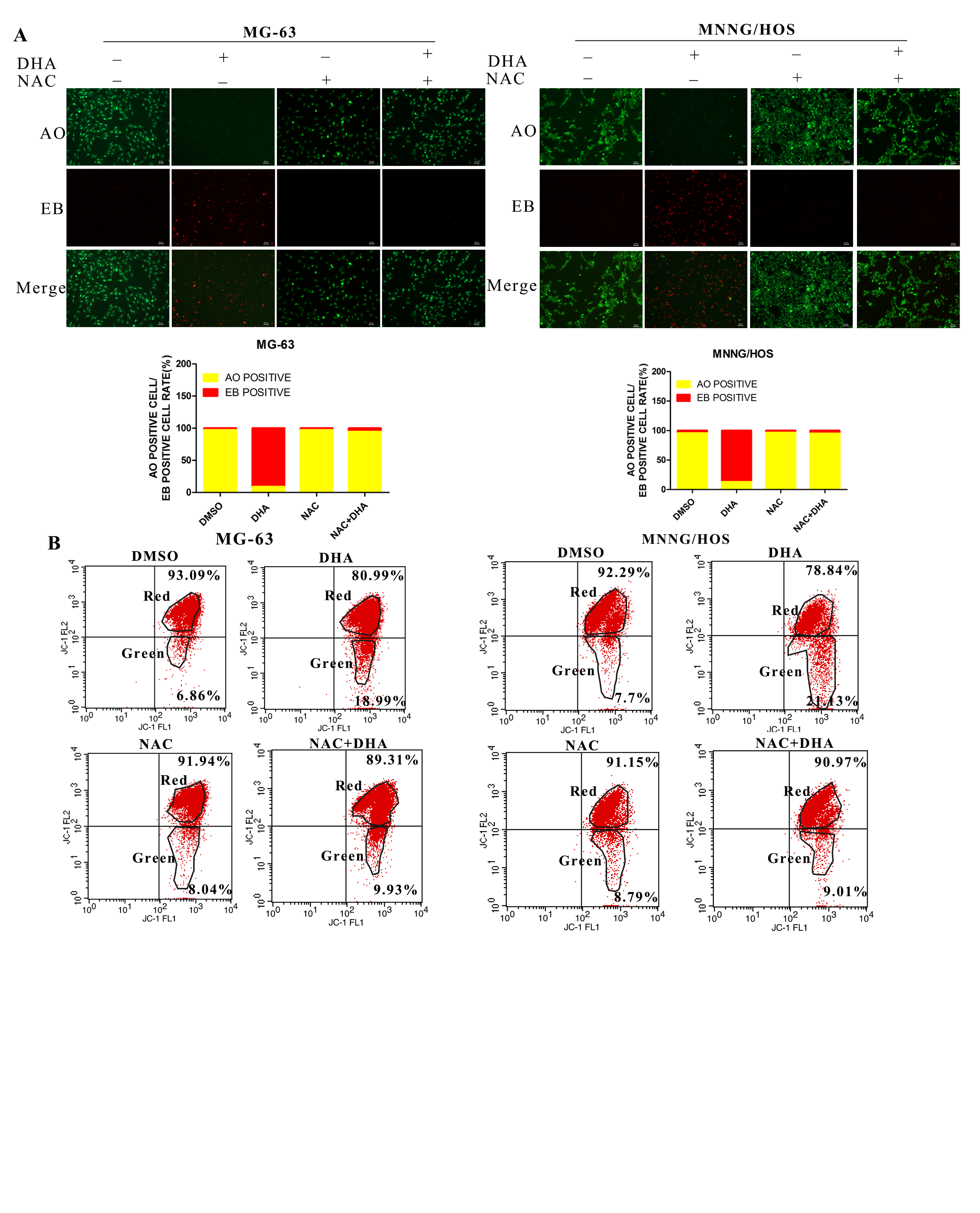

Supplement: Figure S4 — NAC protects osteosarcoma cells from cell death and mitochondrial membrane potential decrease induced by DHA. AO/EB staining of 20μM DHA-treated MG-63 (A) and MNNG/HOS (B) cells, with or without 5mM NAC pre-treatment for 24h. (B) Measurement of mitochondrial membrane potential with JC-1 fluorescent probe and flow cytometry following 20μM DHA treatment for 24h in MG-63 cells, with or without 5mM NAC pre-treatment. *P < 0.05 versus control, **P < 0.01 versus control, ***P < 0.001 versus control. Scale bar = 50μm. [file Image_4.tif]

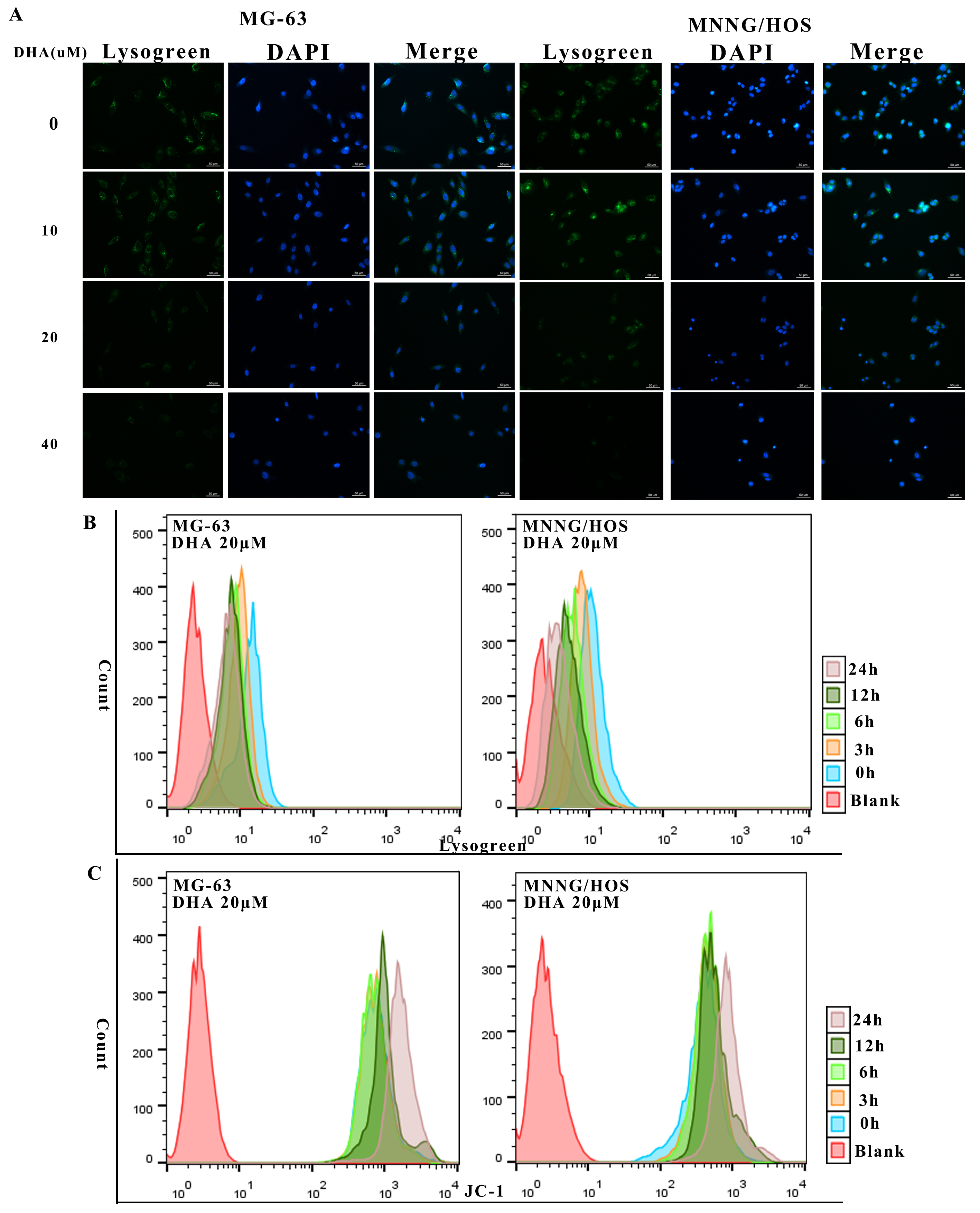

Supplement: Figure S5 — DHA induced LMP and MMP decade. (A) Lysogreen staining of MG-63 and MNNG/HOS cells. Cells were treated with 10μM, 20μM and 40μM DHA for 24h and cells were observed using a fluorescence microscope (n = 3). (B) Lysogreen staining of MG-63 cells and MNNG/HOS cells with 20μM DHA treatment at 0h, 3h, 6h, 12h, 24h were analyzed by flow cytometry. (n=3) (C) JC-1 staining of MG-63 cells and MNNG/HOS cells with 20μM DHA treatment at 0h, 3h, 6h, 12h, 24h were analyzed by flow cytometry. (n=3) Cells were observed with 20× objective. Scale bar = 50μm. [file Image_5.tif]

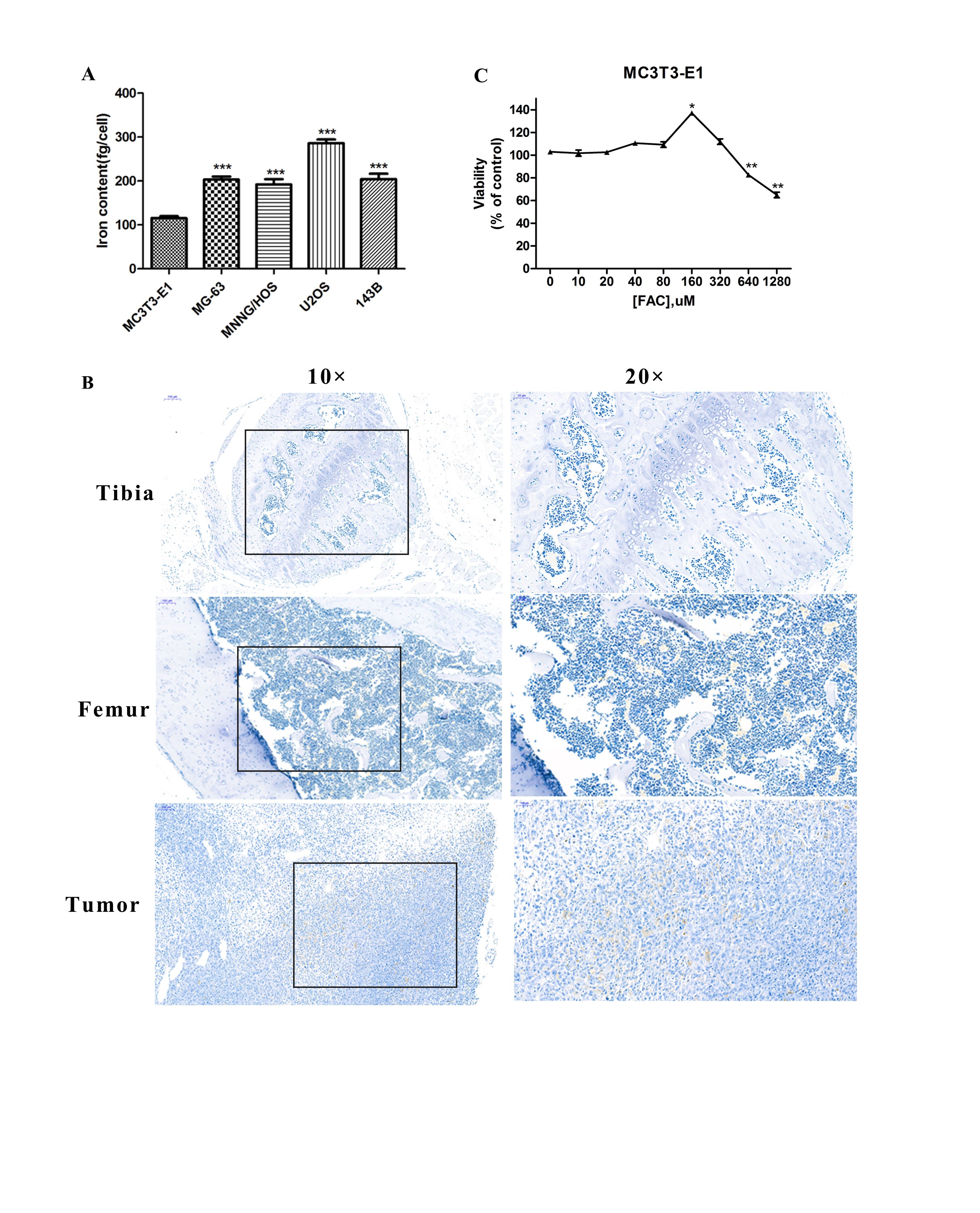

Supplement: Figure S6 — High iron content in osteosarcoma promotes the anti-osteosarcoma properties of DHA. (A) Iron content in non-cancerous osteoblast and osteosarcoma cells. (B) Iron content in mouse tibia, mouse femur and osteosarcoma tissue. (C) Cell viability assays for MC3T3-E1 cell lines treated with FAC at different concentrations. (n=5) *P < 0.05 versus control, **P < 0.01 versus control, ***P < 0.001 versus control. [file Image_6.tif]

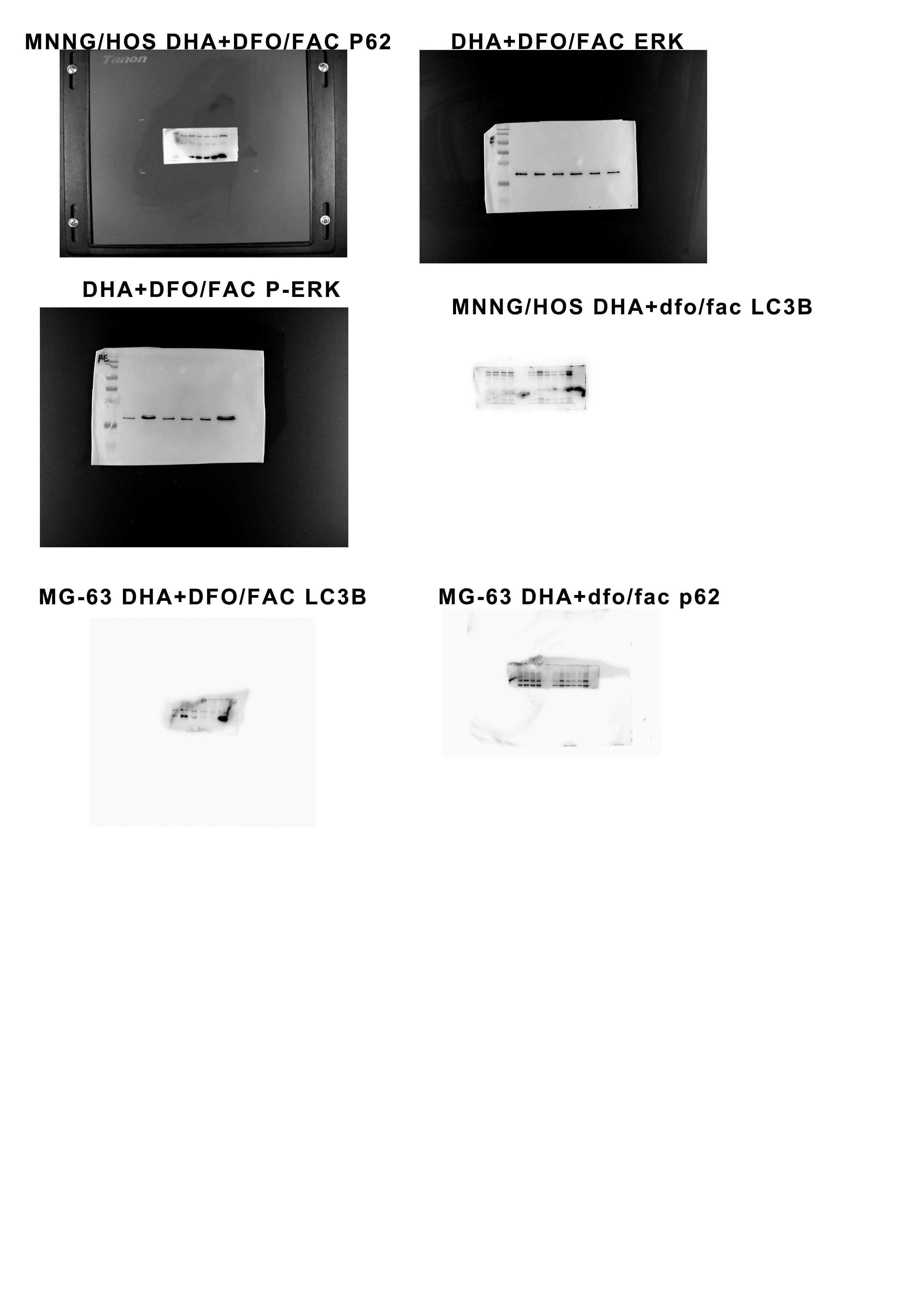

Supplement: Figures S7–S11 — Original image files of the blots included in the article Figures. [file Image_7.tif]

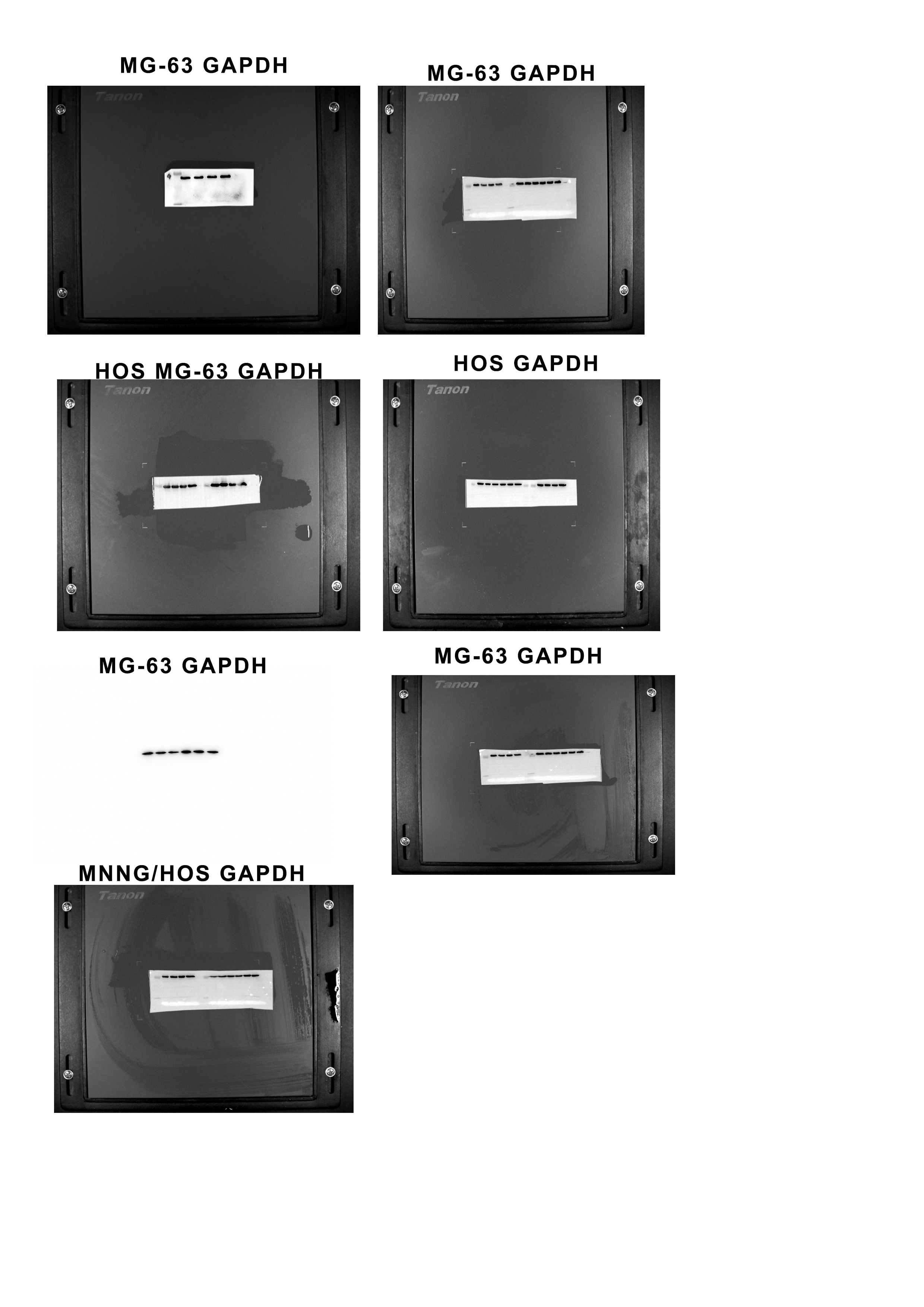

Supplement: Supplementary file 8 [file Image_8.tif]

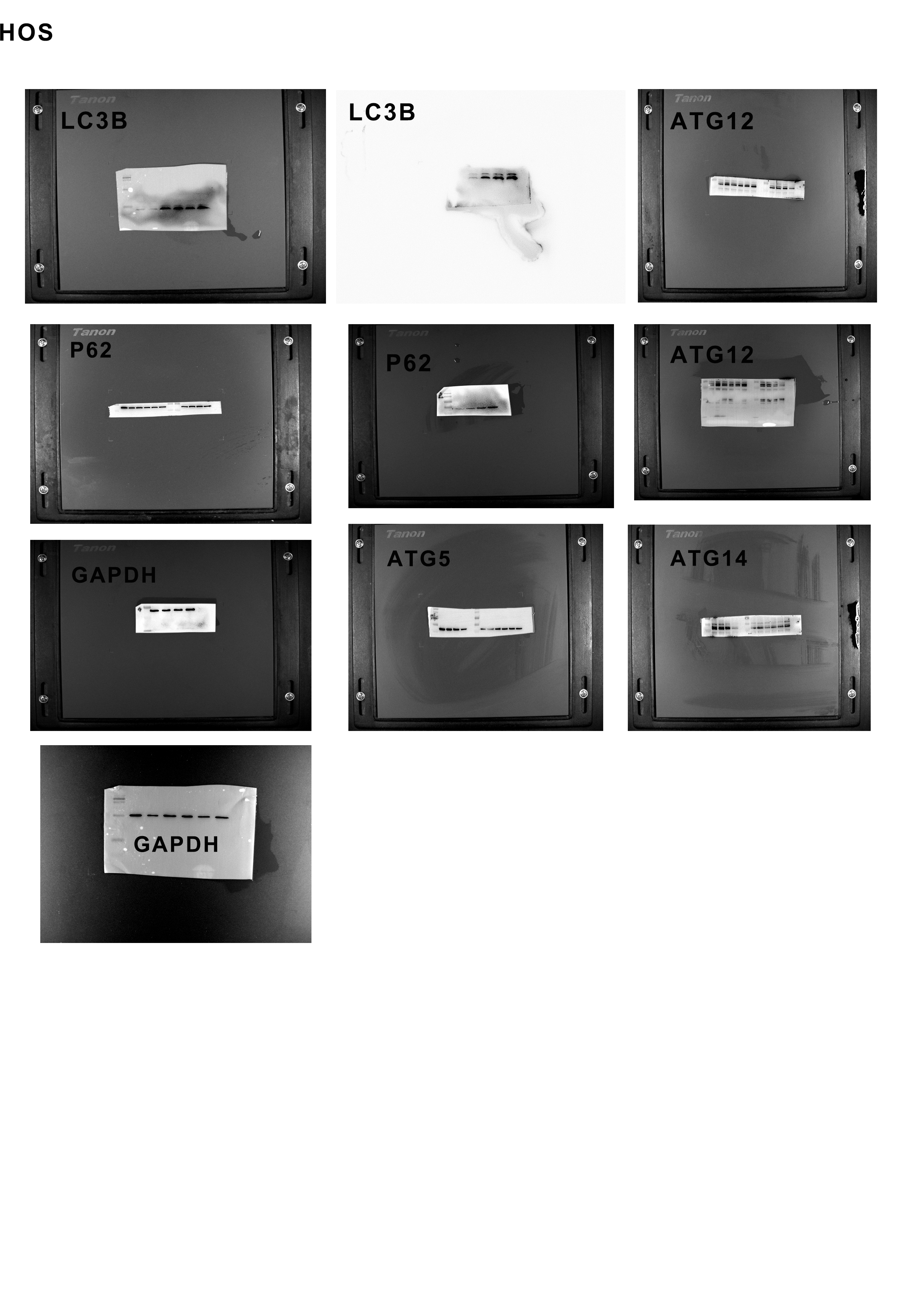

Supplement: Supplementary file 9 [file Image_9.tif]

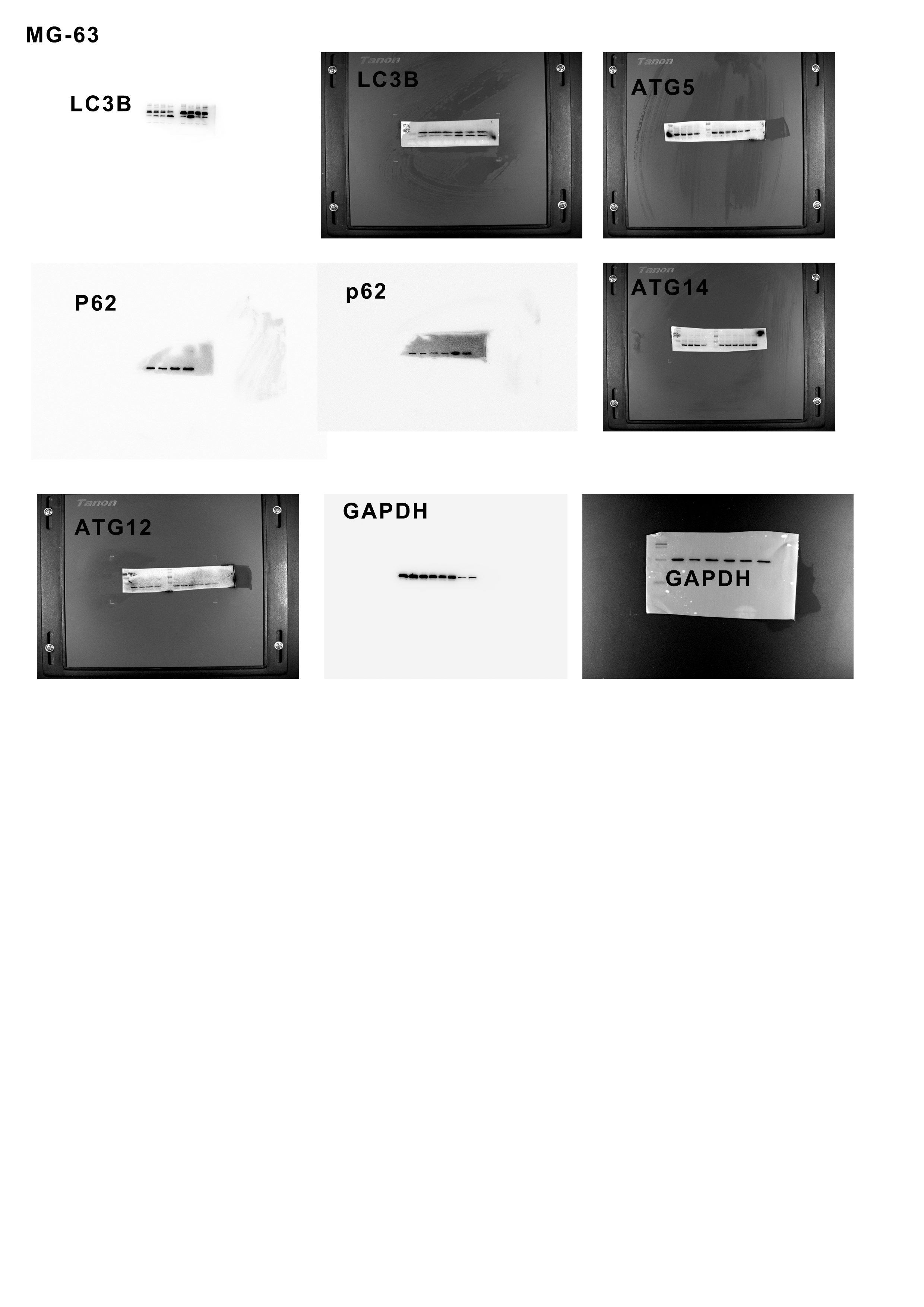

Supplement: Supplementary file 10 [file Image_10.tif]

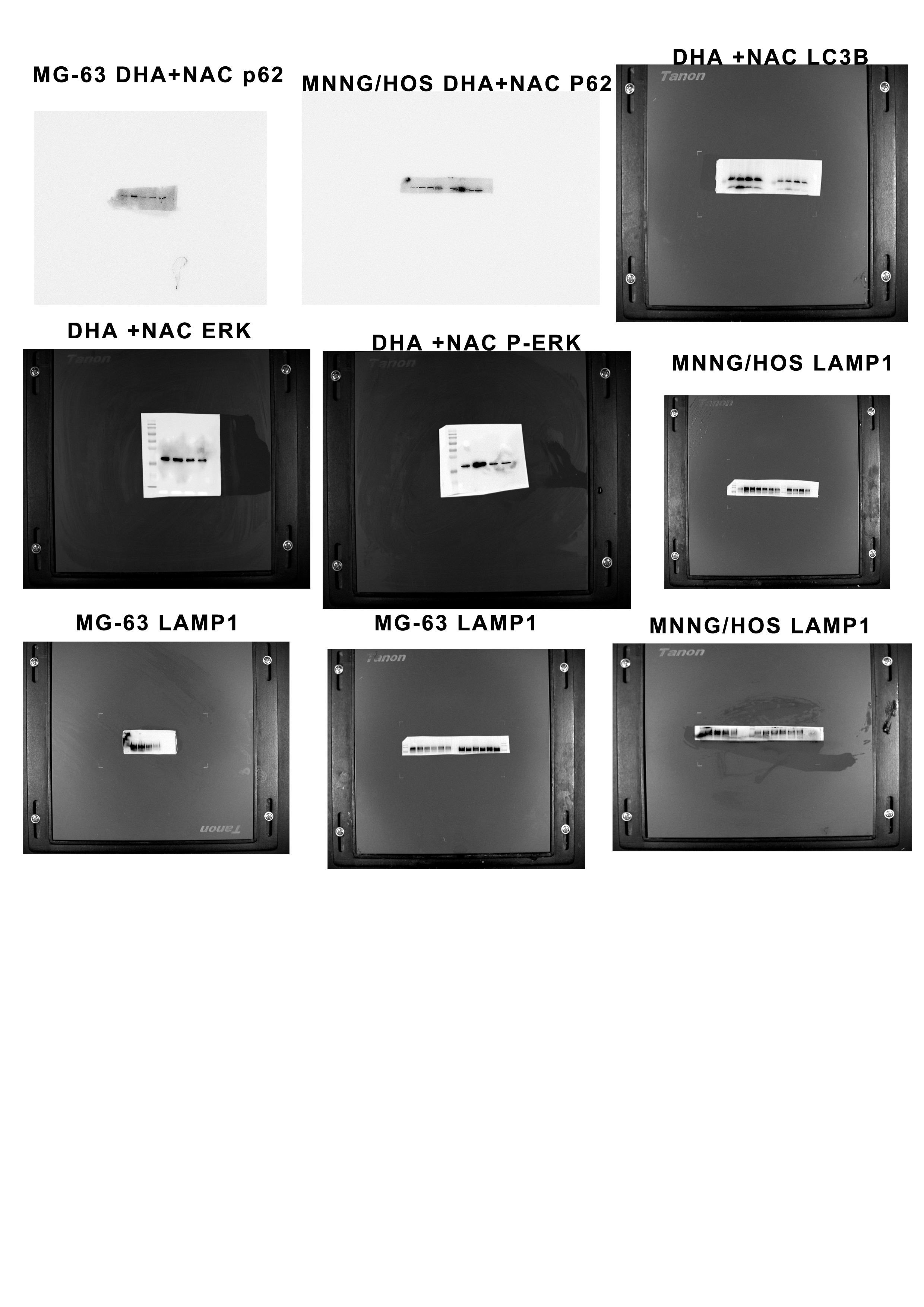

Supplement: Supplementary file 11 [file Image_11.tif]
